# Machine-Learning-Guided Discovery of Electrochemical Reactions

**DOI:** 10.1021/jacs.2c08997

**Published:** 2022-12-02

**Authors:** Andrew F. Zahrt, Yiming Mo, Kakasaheb Y. Nandiwale, Ron Shprints, Esther Heid, Klavs F. Jensen

**Affiliations:** † Department of Chemical Engineering, Massachusetts Institute of Technology, Cambridge, Massachusetts02142, United States; ‡ College of Chemical and Biological Engineering, Zhejiang University, Hangzhou310027, China; § Institute of Materials Chemistry, TU Wien, Vienna1060, Austria

## Abstract

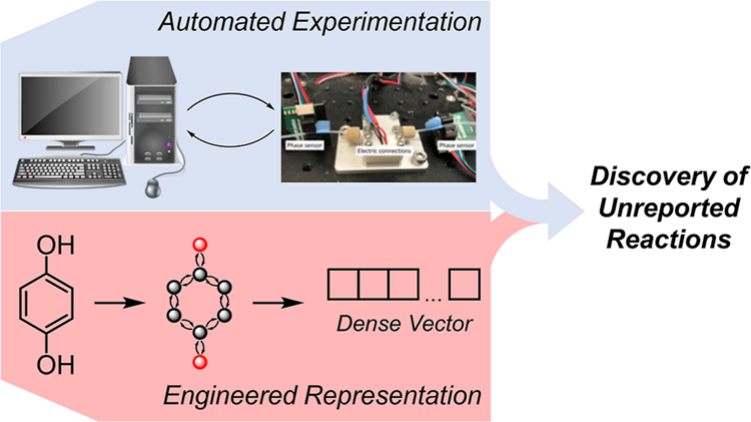

The molecular structures synthesizable by organic chemists dictate the molecular functions they can create. The invention and development of chemical reactions are thus critical for chemists to access new and desirable functional molecules in all disciplines of organic chemistry. This work seeks to expedite the exploration of emerging areas of organic chemistry by devising a machine-learning-guided workflow for reaction discovery. Specifically, this study uses machine learning to predict competent electrochemical reactions. To this end, we first develop a molecular representation that enables the production of general models with limited training data. Next, we employ automated experimentation to test a large number of electrochemical reactions. These reactions are categorized as competent or incompetent mixtures, and a classification model was trained to predict reaction competency. This model is used to screen 38,865 potential reactions in silico, and the predictions are used to identify a number of reactions of synthetic or mechanistic interest, 80% of which are found to be competent. Additionally, we provide the predictions for the 38,865-member set in the hope of accelerating the development of this field. We envision that adopting a workflow such as this could enable the rapid development of many fields of chemistry.

## Introduction

Functional molecules permeate society. From pharmaceuticals and agrochemicals to functional materials with applications in electronic materials, polymers, and nanotechnology, the capacity to access desired molecular functions hinges on our ability to synthesize new molecules. The molecular structures accessible to the synthetic chemist and thus the functional molecules that impact every aspect of our world are limited by the types of molecules which can be synthesized. As such, developing new synthetic methods is of paramount importance as the field increasingly targets unexplored molecular architectures in the search for unprecedented molecular functionality. Driven by this need, workflows aimed at expediting the discovery of chemical reactions have gained popularity in recent years.

Chemical intuition and mental heuristics, along with a good understanding of first principles and mechanisms, have traditionally been the predominant means by which new chemical reactions have been discovered and developed. In recent years, new workflows to simulate serendipitous discovery or unveil unexpected reactivity have gained traction as a means of reaction discovery.^1^ These approaches typically rely on high-throughput experimentation of batch reaction mixtures of a few reactants, with the search domain truncated to reactions with a specific type of catalyst, those with reactants containing a type of desired functionality, or those belonging to a specific mechanistic regime.^2−10^ In general, reactant selection in these studies is subject to a number of factors, including perceived differences in reactivity, compatibility with a certain mode of catalysis, compatibility with the desired analytical technique, and empirically measured reactivity. Despite the success of many of these studies, such approaches to reaction discovery have not achieved mainstream use, with chemical intuition remaining the primary method of reaction discovery in organic synthesis.

We hypothesize that this situation is owed to the vast number of hypothetical reaction mixtures that can exist within certain regions of chemistry. Even if researchers limit the scope of the discovery study and employ high-throughput experimentation techniques, most reactions capable of being assessed reliably is on the order of thousands, whereas the number of hypothetical reaction mixtures is frequently on the order of millions. As such, experimentalists cannot hope to reliably sample all regions of reactivity space and will almost certainly miss the many small but groundbreaking islands of useful synthetic reactivity in a large sea of incompetent reaction mixtures. We propose that using statistical methods and machine learning could provide a solution to this longstanding limitation in reaction discovery campaigns. By using machine learning to inform which reactions are tested on experimental platforms, the overall rate of discovery can be greatly improved in such studies. Further, by using machine learning to direct the discovery process, a more comprehensive survey of the reactivity space is possible. Given the breadth of possible chemical space for reaction discovery, it is reasonable to argue that the implementation of a data science/machine learning workflow is a potential way to effectively navigate this reactivity space.

Applications of computer-guided approaches to reaction discovery are rare when compared with the numerous experimental studies aimed at achieving the same goals. Most early studies in this field never extended beyond proof-of-principle.^11−14^ Recently, Cronin and co-workers have re-invigorated the field of computer-guided reaction discovery, demonstrating the ability to use machine learning to predict productive reactant combinations.^15,16^ This pioneering work uncovered multiple unreported reactions; however, the scope of this approach has not been extended beyond the molecules included in the original survey. No tool exists in which chemists have evaluated tens of thousands of reactants that have not previously been tested. To address this challenge, we have developed a workflow incorporating automated experimentation with a machine-learning-guided protocol for reaction discovery capable of computationally evaluating vast quantities of virtual reactions, including those with reactants not included in the training data. Using this workflow, a chemist can feed entire catalogs of commercially available molecules through a reactivity prediction model and use the predictions from that model to inform which reactants should be purchased and tested experimentally. As a first step toward developing this tool, we have designed a case study aimed at discovering convergent paired electrolytic reactions. Specifically, through feature engineering and modeling, we have explored tens of thousands of potential reactants to identify reactive partners in this field of chemistry.

**Figure 1:**
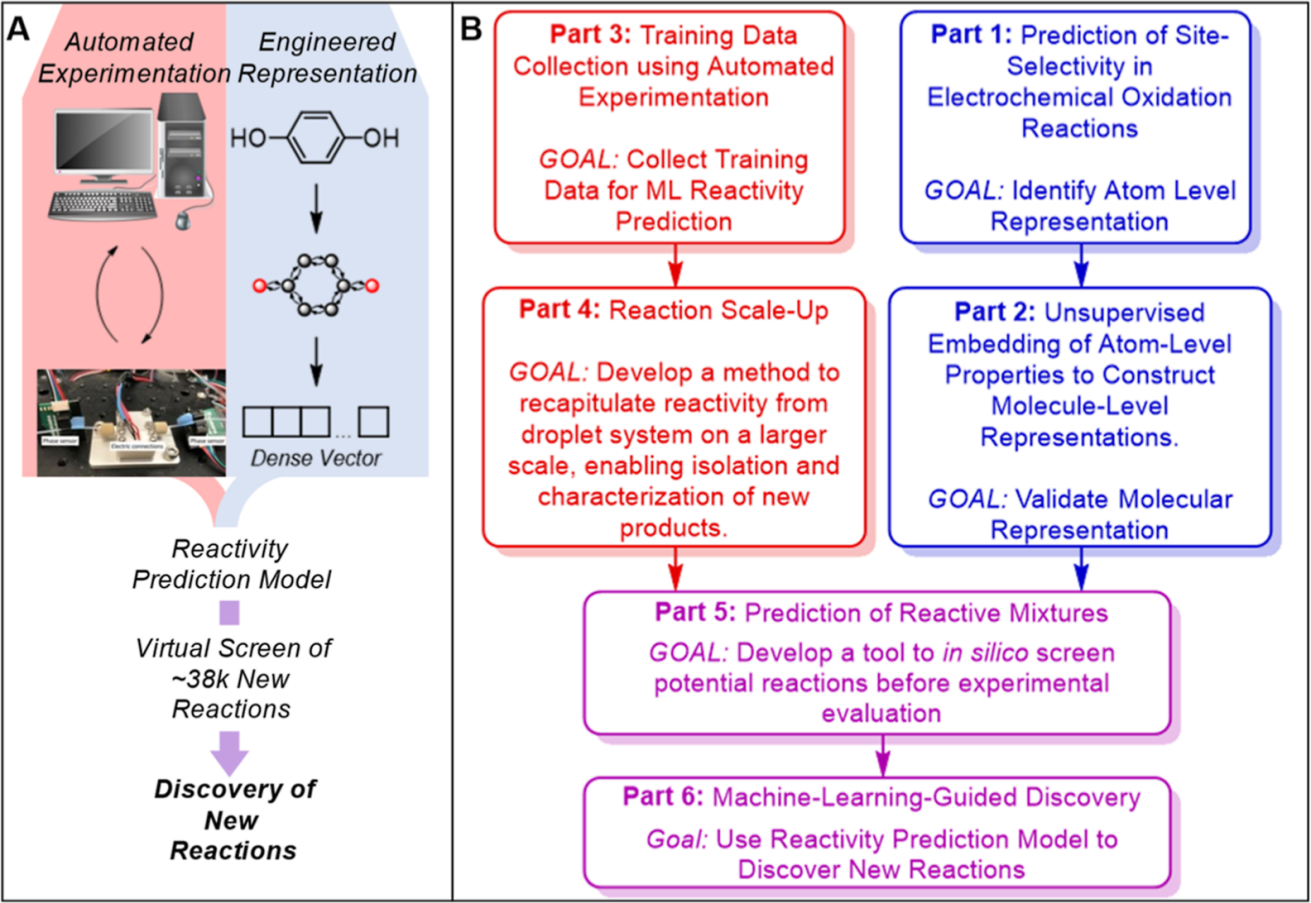
(A) General and (B) detailed overview of the present study. Reactor image in (A) adapted from ref (21). Copyright 2021 John Wiley and Sons.

Synthetic electrochemistry has been identified as a promising, emerging area of organic chemistry owing to (1) the ability to devise a variety of useful disconnections from unfunctionalized starting materials, (2) mild and functional group tolerant reaction conditions, and (3) the tunability of oxidation or reduction by adjusting the potential applied. To approach this problem in the field of synthetic electrochemistry, three challenges must be overcome. The first challenge is one of molecular representation. Because the hypothetical reaction space is large and training data is inherently limited, we reason that it will be necessary to engineer a feature set enabling the construction of more general models using sparse training data. To address this challenge, we have devised an extension of the mol2vec concept,^17^ embedding quantum chemical information in a fixed-length vector. Notably, in the current work, we use this representation in multiple case studies, suggesting broader applications for this representation beyond reactivity prediction in electrochemistry. The second challenge is data collection; the throughput of experimentation is likely too low using conventional batch electrochemical reactors. To achieve this task, our laboratory has developed a microfluidic platform for screening many electroorganic reactions rapidly with small quantities of reagents, overcoming the inherent limitation of sequential batch screening, thus constituting an ideal modality for reaction discovery in the field of organic electrochemistry.^18−21^ Finally, the third challenge is to use the dataset and the representation to construct models, which can then be used to discover reactions. For this, we have devised a “chemist-in-the-loop” workflow for the selection of new reactants using predicted outcomes and prediction probabilites to inform which reactants to test next. Using these components, we have successfully devised and implemented a proof-of-principle workflow for reaction discovery, unveiling multiple, which we believe warrant further investigation on the basis of synthetic or mechanistic interest (Figure 1A). We believe this work is a strong first step toward machine-learning-enabled reaction discovery.

To guide the reader through the logic of the following studies, we provide a more detailed overview in Figure 1B. To reiterate, the overarching goal of the study is to use machine learning to discover unreported transformations, defined in this study as reaction templates that have not been previously reported. This was evaluated by performing a forward substructure search for that reaction in SciFinder. To achieve this, we reasoned that a general representation containing relevant electronic structure information would yield best results. As such, we constructed this representation by first considering which atomic properties were relevant. In order to do this, we executed Part 1: Prediction of Site Selectivity in Electrochemical Oxidation Reactions. In this section, we successfully create a model capable of predicting if an atom will be oxidized in the course of the reaction. This model only considers atomic properties—each atom is an individual input. With the success of part 1, an adequate atom-level representation was identified. However, for the desired task of predicting new reactivity, a molecule-level representation was required. As such, in part 2, we devise a method of combining the atom-level representations into molecule-level representations. These molecule-level representations are validated by comparison with other 2D-representations for multiple tasks. With a representation identified, experimental data was gathered with an automated platform (part 3). To ensure it is possible to characterize the discovered reactions, a procedure was developed to scale up the droplet reactions using a recirculating system with an electrochemical flow cell (part 4). With training data from part 3, a scale-up and analytical workflow from part 4, and a molecular representation from part 2, we were then able to construct a reactivity prediction model (part 5). This model was used to evaluate 38k reactions *in silico*, and then used to select new reactions to evaluate experimentally (part 6). These reactions were tested, scaled up, and evaluated to verify new reactions. It should be noted that the goal here is to simply unveil the reactivity of new reactive partners; this method provides the first hit rather than an optimized synthetic method. In this process, we have discovered a number of unreported new reactions.

## Results and Discussion

### Part 1: Prediction of Site Selectivity in Electrochemical Oxidation Reactions

**Figure 2:**
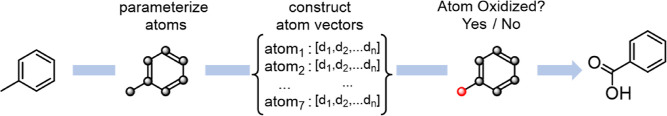
Workflow for electrochemical oxidation, part 1. Features for atoms are calculated, and the individual atoms are parameterized. The ML model is a binary classification model predicting if an atom will or will not be oxidized.

As described previously, the first step toward developing a generalizable molecular representation was to identify suitable atom-level features that could be embedded into a molecule-level representation. To achieve this goal, we reasoned that developing the representation using literature data for a related modeling task would enable this work with minimal experimental overhead. Because the objective of the current study was to identify competent electrochemical processes, a prototype dataset of electrochemical oxidation reactions was selected. To simplify the task of determining which atom is oxidized, we only considered reactions in which one or more carbon atoms are formally oxidized. With these constraints, a dataset of electrochemical oxidations was collected, containing 370 reactions which were manually curated from Reaxys. The dataset has 5485 non-hydrogen atoms, 453 (8%) of which were oxidized during the transformation. The task selected was to individually classify each atom as either one that is oxidized in the reaction or one that remains unchanged in the reaction (Figure 2).

**Figure 3:**
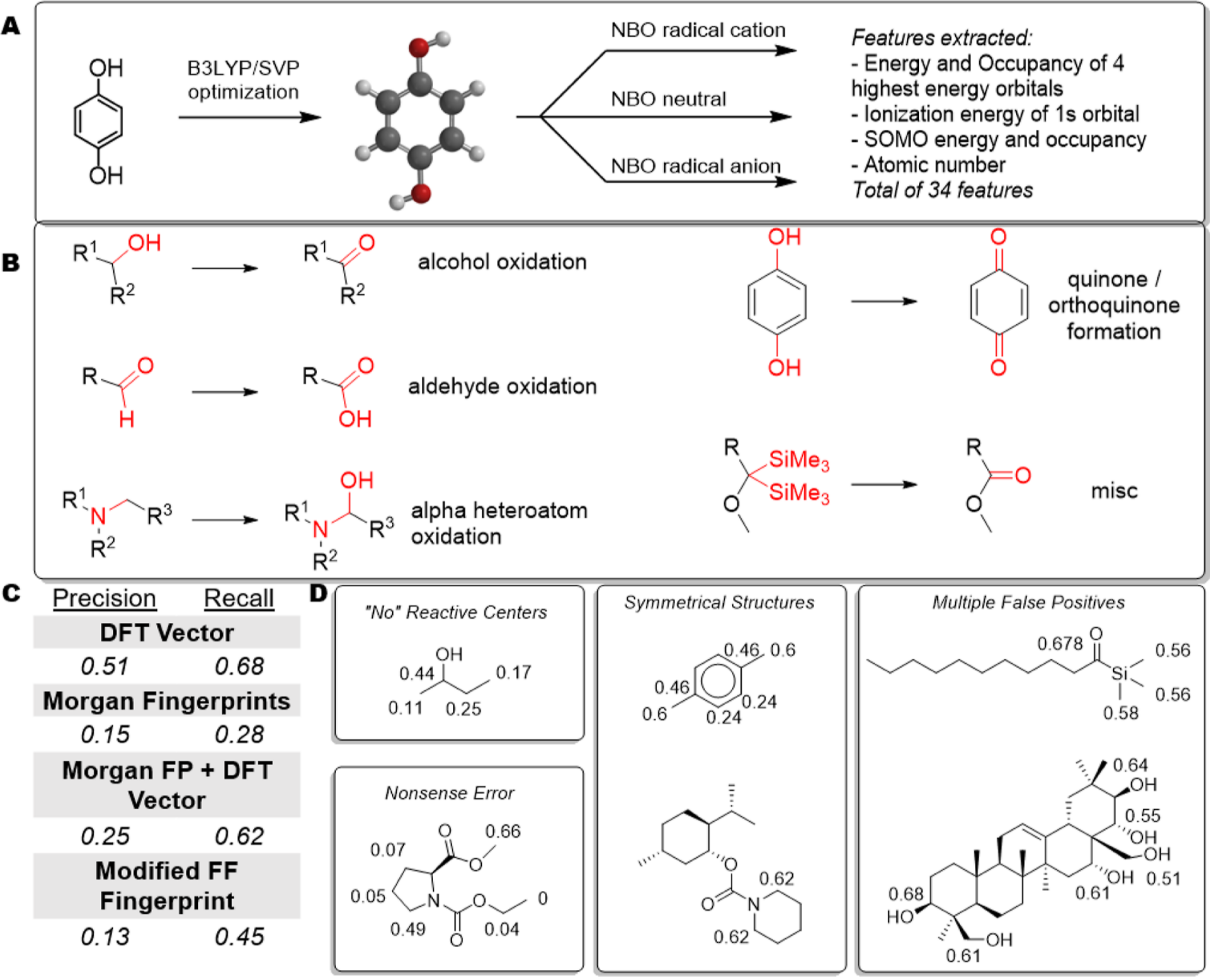
(A) Workflow for descriptor calculation. (B) Templates used in the leave-one-group-out approach. (C) Modeling results using the DFT vector, Morgan fingerprints, Morgan fingerprints concatenated with the DFT vector, and the fingerprint from ref (24) calculated from the aggregate of left out groups. Precision corresponds to the proportion of “positive” (in this case, “is oxidized”) predictions that are correct, and recall corresponds to the portion of observed positives that are correctly identified. (D) Selected types of errors identified during manual error analysis. The numbers indicate prediction probabilities for the corresponding atoms.

To develop the representation, a 34-dimensional feature vector was constructed for each atom using quantum chemical data from natural bond orbital calculations (Figure 3A). This vector contains occupancy and energy values for different atomic orbitals for the neutral, oxidized, and reduced analogues of a given molecule. Although this process is quite time-intensive, our hypothesis is that by developing an excellent molecular representation, more general models can be constructed with less experimental data. As such, experimental overhead is reduced which should results in a net reduction of time and resources.^22^ The performance of this representation was compared to different models using Morgan fingerprints^23^ and an adapted atom-level representation from ref (24) as baseline models.

In order to rigorously test the generalizability of the models, a leave-one-group-out approach was implemented. In this approach, the data were partitioned by reaction template (Figure 3B). Four reaction templates were used to construct the model, while the fifth reaction template was held out as an external test set. This process was repeated until each template group was held out, and the aggregate scores were reported as the performance metric (Figure 3C). In this way, the model is forced to predict outcomes for reaction types that are absent from the training data; thus, this design gives a better indication of which descriptor sets give more general models. Using this design, the density functional theory (DFT) feature vector significantly outperforms baseline models using alternative methods, suggesting that this approach produces a more generalizable model than the alternatives. Additionally, it was also observed that concatenating Morgan fingerprints and the DFT vector also gave suboptimal results. It is likely this is a result of the much higher dimensionality of the fingerprint with respect to the DFT vector.

The small dataset size made it reasonable to manually examine the erroneous predictions and assess the types of errors encountered. Interestingly, many of the erroneous predictions seem to be rooted in a chemical basis (Figure 3D). Although some predictions have been labeled as “nonsense errors,” or errors which have no chemical basis (Figure 3D, lower-left), many are chemically interpretable. For example, some structures are predicted to have no reactive centers (Figure 3D, left). However, if the prediction values are examined, in many instances, the center with the highest probability of being oxidized is the correct center. In other occurrences, the reactants are symmetrical compounds (Figure 3D, middle-right). In this case, it is possible that more than one center is equally likely to be oxidized and that during the reaction only one center is. As such, both centers are correctly identified as oxidizable centers. However, because only one gets oxidized, the other counts as a false positive in the scoring function. In this dataset, there are 30 such examples of false positives. Another case similar to errors in symmetrical structures is the scenario of many false positives (Figure 3D, right). In this circumstance, the model identifies centers that could hypothetically be oxidized. However, only one or some are oxidized in the reaction. Interestingly, in many cases, the rank-order of which centers should be oxidized is in line with the chemical intuition. An extreme example of this phenomenon is shown in Figure 3D, bottom right. Therein are many oxidizable centers and with increasing reaction time and potential one would expect multiple centers to be oxidized even though only one is oxidized in this case. This example is interesting because the oxidation probabilities are in line with chemical intuition in that the more electron-rich centers are oxidized first—the secondary centers have higher oxidation probabilities than their primary analogues, and the “right” half of the molecule with more inductively withdrawing groups has lower oxidation probabilities than the “left” half of the molecule. The secondary alcohol with the highest oxidation probability (0.68) is indeed the center to be oxidized experimentally. Given the success of these models for this challenging task and the intuitive behavior that tracks well with chemical intuition, we reasoned that the atom-level features identified would be suitable for tasks related to electrochemical processes. Accordingly, we next moved on to using this data to construct molecule-level representations.

### Part 2: Unsupervised Embedding of Atom-Level Properties to Construct Molecule-Level Representations

To convert the atom-level representation used in the previous study to a molecule-level representation used for whole-molecule predictions, the atomic properties were transformed into a molecular feature vector using the graph2vec process.^25^ This process is similar to the mol2vec concept and uses the atom level features as node attributes in a graph, which is embedded into a fixed-length vector.^17^ However, in this case, the node attributes are properties derived from quantum chemistry. Our hypothesis is that the physical basis of the representation will enable this approach to produce more general models. It is worth noting that this approach is inspired by a related approach in which quantum chemical properties are used to augment learned representations to make more accurate predictions.^26,27^ However, in those applications, the learning procedure is supervised and the application space is typically limited to a well-defined region of reactivity space. In this work, the goal was to generalize in reactivity space in a way that is more agnostic to mechanism or the overall transformation. Further, it may be desirable to have an unsupervised analogue to enable exploration of reactivity space without labeled points. As such, we reasoned an unsupervised embedding process could yield a representation fitting these requirements while retaining the benefits of including quantum chemical features.

**Figure 4:**
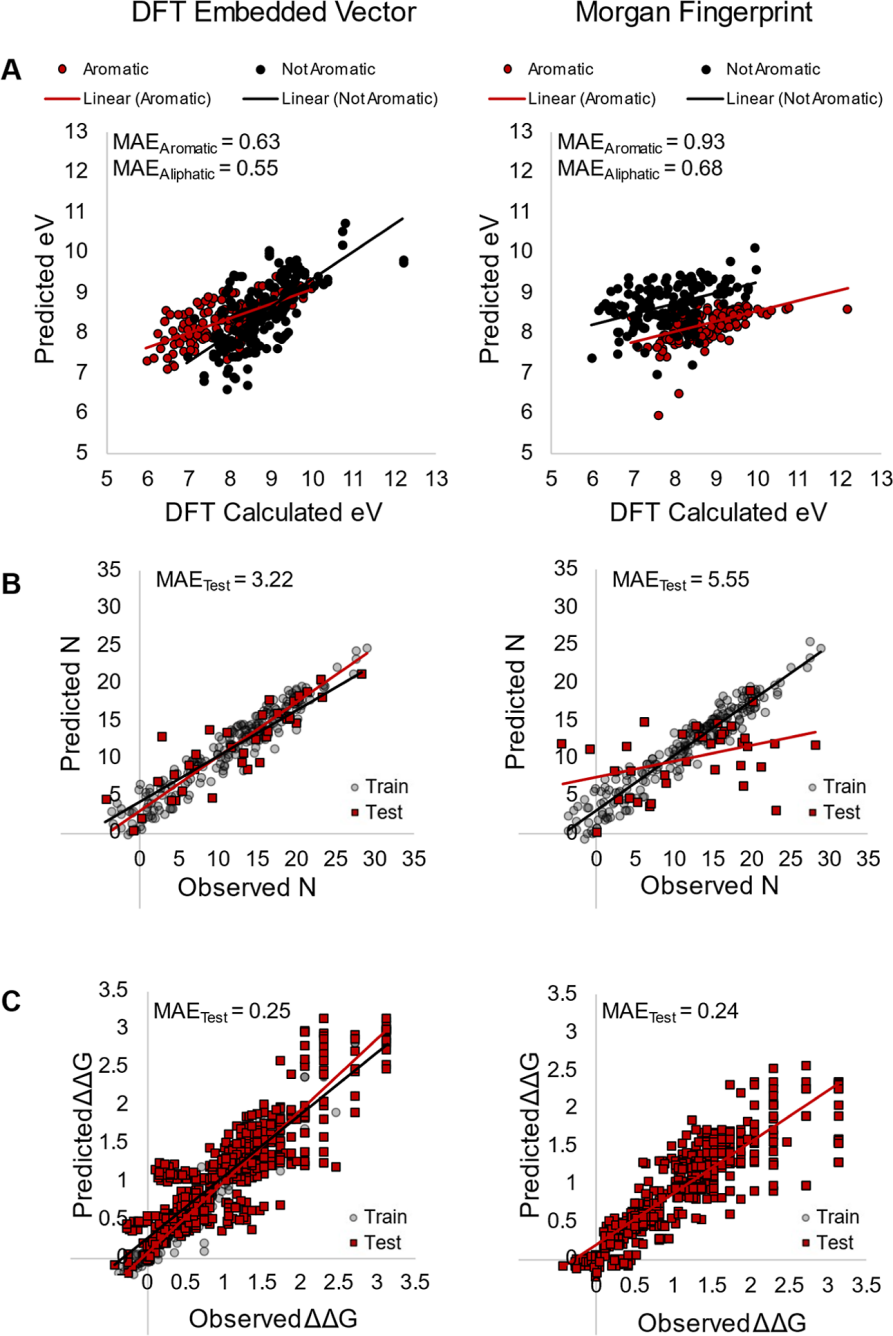
Comparison of performance of embedded DFT representation and Morgan fingerprints for (A) ionization potential prediction, (B) nucleophilicity prediction, and (C) enantioselectivity prediction.

As a first test of this hypothesis, a model predicting the ionization potential of molecules in the previous 370-member dataset was generated and compared to baseline models employing Morgan fingerprints. Specifically, the ionization energy for each molecule was calculated, and the set was divided into aromatic molecules (158 in total) and nonaromatic molecules (212 in total). The projection to latent structure models was then trained on either the aromatic molecules or the nonaromatic molecules and used to predict the ionization potentials. In this task, the fingerprints generated from molecular graphs with quantum chemical features outperformed the Morgan fingerprint baseline model, validating this approach for devising molecular representations (Figure 4, top).

To further assess the generalizability of this representation, it was used to predict the experimentally measured nucleophilicity and enantioselectivity values of a set of compounds. For nucleophilicity, a dataset containing 341 unique nucleophilicity parameters was collected from the literature.^28^ This dataset was used in a related study in which features engineered for that application were used for accurate model generation. In this case, the embedded graph representation also outperforms Morgan fingerprints substantially (Figure 4, middle).

For the enantioselectivity dataset,^29,30^ the embedded graph representation performs similarly to Morgan fingerprints (Figure 4, bottom). A reasonable explanation for the similar performance of these two representations is that both intrinsically use 2D molecular representations. Even though the DFT-embedded fingerprint contains quantum chemical information, it does not directly reflect the 3D shape of the molecule. As such, it is unsurprising that it performs similarly to other methods derived from 2D molecular graphs. In contrast, because the representation contains information pertaining to electronic structure, it does outperform Morgan fingerprints when the prediction task is more closely related to the electronic structure (ionization potential, nucleophilicity). As such, we believe this representation will have broader implications beyond the scope of this study. With this representation in hand, we then moved on to applying it to the main goal of this study that is the discovery of new electrochemical reactions.

### Part 3: Training Data Collection Using Automated Experimentation

**Figure 5:**
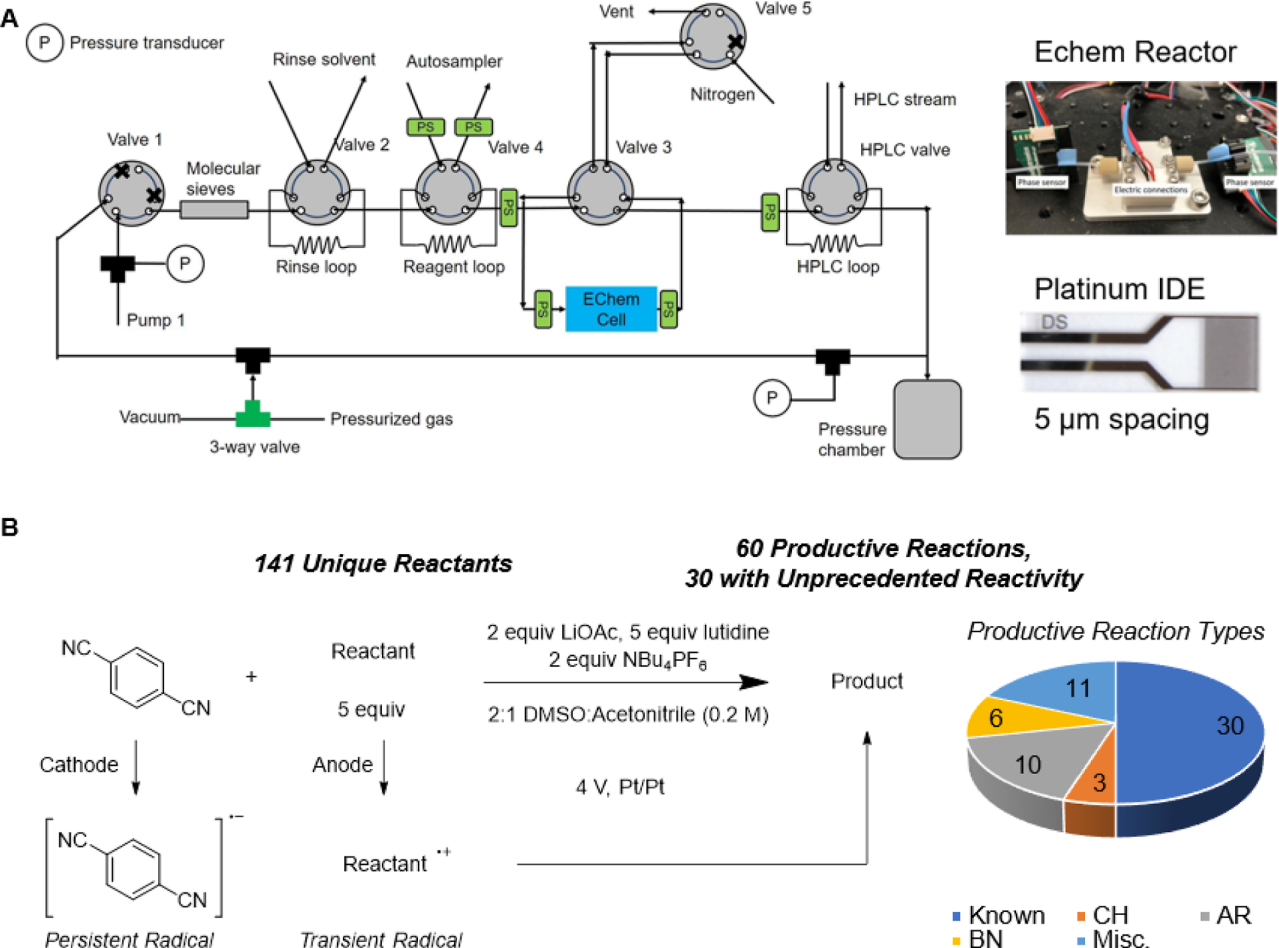
(A) Automated experimentation platform and (B) reaction system and distribution of productive reactions. Reactor image in 5A adapted from ref (21). Copyright 2021 John Wiley and Sons.

With an adequate featurization of molecules identified, the next step of the process was to collect training data experimentally, which then served as training data for the desired reactivity prediction model. The reaction studied in this system was the reaction of different potential reactants with 1,4-dicyanobenzene (DCB) in a convergent paired electrolytic reaction (Figure 5B). In this reaction, DCB is reduced at the cathode to form a persistent radical anion. If the reactive partner being probed is competent, it should be oxidized at the anode to form a radical anion, which then couples with the DCB anion to form the product. If the anode half reaction is not competent, DCB will not convert because there is no counter anodic reaction; as such, the competence of the reactive partner can be assessed by monitoring the conversion of DCB. Because of the expected modeling challenge associated with predicting novel reactive partners, a fixed set of reaction conditions was devised that generally worked for known reactants in convergent paired electrolytic reactions with DCB. In this way, the modeling challenge is simplified to a binary classification model tasked with predicting conversion or no conversion and does not have to learn the interaction between reaction conditions and reactant structure to predict competence. Clearly, this introduces the limitation of potentially missing some reactive partners that would be competent under different conditions; this was deemed an acceptable limitation for a preliminary study. Tuning the numerous parameters of electrochemical reactions (e.g., solvent, electrolyte, current/potential, electrode material, other discrete variables, etc.) is a problem unto itself and a future direction we are eager to explore.

Some discussion is necessary with regard to the selection of this model system. The choice of DCB for use in discovery campaigns was inspired by the work of MacMillan and co-workers exploring the concept of “accelerated serendipity.”^2^ Having been used in a reaction discovery campaign previously, and being well established to be electrochemically active,^21^ it was reasonable to expect that some reactions involving DCB could be discovered through random selection (e.g. without the machine learning workflow). This choice is critical to the evaluation of the workflow developed in this work. When developing model systems for new ML-workflows, it is important to pick one in which the outcome would be interpretable in any outcome of the study. By choosing this substrate, we know we should find new reactions even by random sampling (note: we approximate random sampling in our collection of training data). If the machine-learning workflow fails to discover new reactions in this area of chemical space (or discovers new reactions at the same rate as randomly sampling), the workflow clearly does not work, and we have not succeeded in our goal of creating a workflow. However, if the machine-learning-guided selection process results in a significantly greater increase in the discovery rate, the workflow is a success. In contrast, if a completely unprecedented area of chemical space was selected, failure to discover new reactions would give an ambiguous result—does the workflow fail, or is the region of chemical space not active? As such, to develop this workflow, selecting a well-established choice was a necessity. Further, even though the cathode half of the reaction is well understood, unintuitive anodic processes and overall transformations can still be discovered even in this well-precedented region of chemical space.

The dataset for the discovery process was collected on the platform depicted in Figure 5A. Reaction droplets are prepared in an automated liquid handler encased in a plexiglass box purged with nitrogen. Inert gas is injected into the line before and after the droplet, and the droplet is transferred to valve 4 in Figure 5A. When the inert gas on either side of the droplet is detected by phase sensors on either side of the valve (signifying the droplet is loaded in the sample loop), the valve switches and the droplet is sent to the electrochemical cell (via valve 3) using a syringe pump filled with argon. The droplet is then oscillated through an electrochemical reactor containing a platinum interdigitated electrode (IDE) with 5 μm interelectrode spacing. The droplet is oscillated for a set reaction time and is then transported to the HPLC for analysis. The system is then rinsed multiple times with solvent, purged with high-pressure gas, and the next reaction is injected.

In this reaction system, a set of 141 readily available molecules were tested (complete list is available in the Supporting Information). The molecules selected were simply molecules available in our laboratory. Although sampling strategies may have an important impact on the workflow, we envisioned that most experimentalists would be interested in this approach to an initial training set as it represents the lowest cost initial investment. Notably, many of the selected molecules were known to work in this reaction; this was an intentional design element to ensure a balanced dataset suitable for the construction of a classification model after the data set is collected.

The initial 141-member set was tested on the experimental platform using reaction conditions that generally worked for known reactive partners. Of this set, 60 molecules resulted in productive reactions, and 81 molecules resulted in unproductive reactions. Of the 60 reactions observed to give appreciable conversion of DCB, 30 contained functional groups already known to be reactive under these reaction conditions (a full discussion on how conversion was assessed is provided in the Supporting Information). The remaining molecules were divided into four groups: (1) electron rich arenes or arenes with large π-surfaces (AR), (2) molecules with benzylic functionality (BN), (3) α-heteroatom C–H containing molecules (CH), and (4) a miscellaneous category (Misc.). A breakdown of reactive mixtures in the initial set is represented in Figure 5B.

### Part 4. Reaction Scale-Up

**Figure 6:**
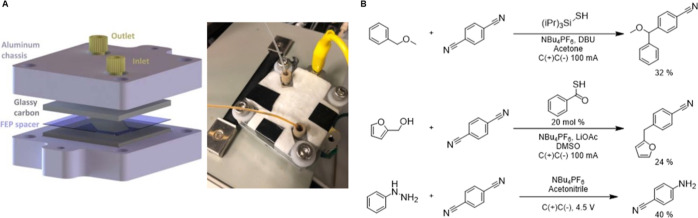
(A) Flow cell used in recirculating reactions and (B) selected examples of reactions discovered while collecting training data. (A) (left) Adapted with permission from ref (20). Copyright 2020 American Association for the Advancement of Science.

In order to rigorously evaluate newly discovered reactions, the droplet system needed to be scaled up to enable isolation and full characterization of the product(s) formed. To develop this process, we first considered some new reactions discovered in the process of collecting training data (i.e., part 3). As described in the previous section, many of the reactions constitute new transformations. As such, we endeavored to evaluate a subset of these reactions on scale to determine the reaction outcome and obtain isolated yields. Preliminary studies using batch reactor setups typically did not result in the same performance as observed in the droplet system (see Supporting Information for details). This is unsurprising, given the small interelectrode distance of the IDEs on the droplet system. As such, a recirculating reactor system was devised to obtain the benefits of the flow cell (good mixing, small inter-electrode distance, high electrode surface area) while also being able to scale up the reaction mixture. This system consists of a parallel plate reactor with a FEP spacer between electrodes, which forms the reactor chamber (Figure 6A). Either glassy carbon or nickel electrodes were used (for a general scale-up procedure, see Supporting Information). The reaction mixture is prepared in a glass culture tube containing a stir bar and fitted with a septum cap and prepared with the typical Schlenk technique. It is then removed and an argon balloon is added. The inlet and outlet of the system are then both fed through the septum and into the solution. The system consists of a Vici Valco M6/M50 positive displacement pump which draws up the liquid and pumps it through the parallel plate reactor, depositing it back into the culture tube. The reaction mixture can then be stirred and run like a typical batch reaction.

Representative examples from each of the categories in Figure 5B were scaled up to isolate and characterize the products formed. Notably, reaction conditions were roughly and empirically optimized during scale-up to ease isolation and purification. The goal of this rough optimization was to ease characterization; current work is underway to more rigorously optimize reaction conditions to convert the discovered reactions into synthetically useful transformations. Three of the discovered reactions were selected as examples of synthetic or mechanistic interest, which are highlighted in Figure 6B. The first is a benzylic functionalization of benzyl methyl ether. The second is the arylation of furfuryl alcohol. Notably, this reaction does not appear to be a C–H functionalization reaction. Rather, we postulate that furfuryl alcohol undergoes an acid-catalyzed dehydration reaction to form an oxocarbenium ion, which is captured by DCB. In this mechanism, the reaction is likely not a convergent paired electrolytic reaction but rather is balanced by a sacrificial reaction at the anode. The third reaction is the amination of DCB with phenyl hydrazine. Notably, no aniline is detected as a byproduct of this reaction, as one might expect if one were to propose a mechanism for this reaction. As such, it represents a reaction of potential synthetic utility that may have been difficult to design rationally.

### Part 5. Prediction of Reactive Mixtures

With a method developed to fully evaluate new reactions, we began work on developing the reactivity prediction model. To do this, the 141-member dataset collected in part 3 was used to train and validate the reactivity prediction model using the molecular representation from part 2. Specifically, 10 different binary classification models for reactivity prediction were created by randomly partitioning the part 3 dataset 10 times, with 116 data points used for training and cross validation and 25 data points used as an external test set. The best model type tested was a Ridge classifier implemented with SKLearn (for a full comparison of models, see Supporting Information); each of the ten models returns an output of 1 or −1, which is averaged to give the prediction for the ensemble. Models employing Morgan fingerprints were also constructed as a baseline for comparison. Notably, the hyperparameters of the unsupervised embedding process of the DFT fingerprint were not adjusted—the same parameters used for ionization potential prediction were used with no further optimization, whereas the length and radius of the Morgan fingerprints were systematically evaluated. Overall, the embedded DFT fingerprint outperforms the baseline model substantially, with an accuracy of 74.4% *versus* 56.2% (Table 1).

**Table 1:** Reactivity Prediction Model Comparison

	partition number^a^										
features	1	2	3	4	5	6	7	8	9	10	mean
DFT embedded vector	76%	76%	80%	80%	64%	76%	68%	80%	80%	64%	74%
Morgan fingerprint (best)	60%	68%	56%	40%	72%	68%	62%	52%	64%	60%	60%

a Accuracies for each partition are reported as percentages.

### Part 6. Machine-Learning-Guided Discovery Workflow

Having identified a method to scale up newly discovered reactions reliably, we sought to develop a workflow using the reactivity prediction model to discover new reactions. To accomplish this task, the following workflow was devised:

(1) A large set of molecules were taken from the GDB17 database and manually augmented with molecules containing other functional groups (see Supporting Information for full details); the total dataset size is 38865 molecules
(2) Each molecule was submitted to the same descriptor calculation protocol as those above, calculating DFT-embedded fingerprints for each
(3) The molecules are fed through the classification model and assigned reactivity predictions and prediction probabilities
(4) The reactions are sorted by prediction probability, and a set of high-probability predictions of synthetic interest are manually selected on the basis of synthetic interest,
(5) Commercially available analogues to the selected molecules are identified, fed through the descriptor calculation protocol, and fed through the classification model to verify that the analogues are indeed competent. In total, 20 molecules were selected and tested.

**Figure 7:**
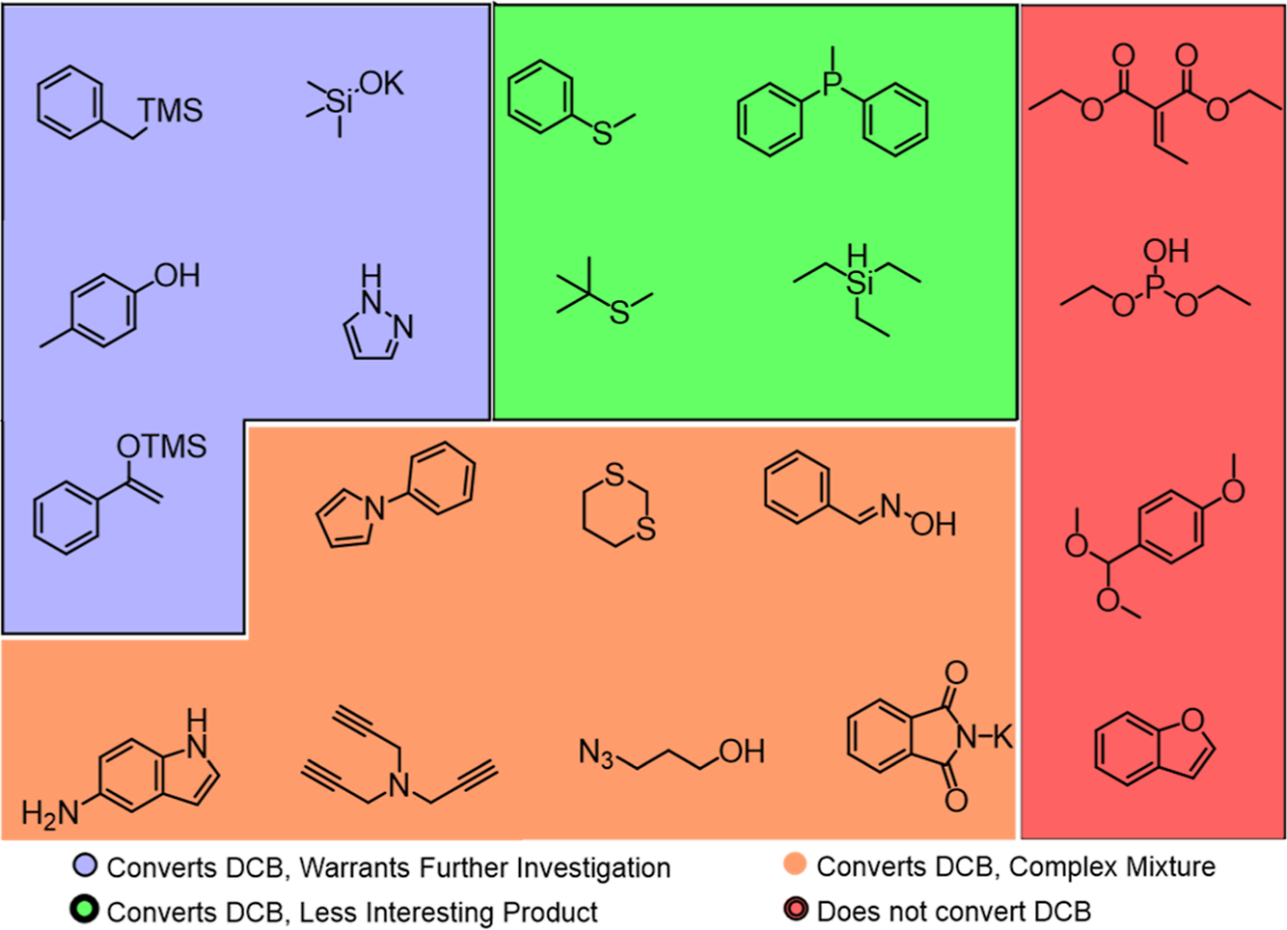
Prediction set of molecules evaluated experimentally.

At this stage, the molecules are purchased and tested experimentally. Notably, this workflow enables rapid prescreening of >38k molecules prior to experimentation; evaluating so many molecules using other methods would be impossible. In total, 38,865 molecules were prescreened, with 824 being returned as likely reactive. The molecules tested via this workflow are depicted in Figure 7.

Examining the 20 molecules selected using the machine-learning-guided workflow, it is notable that 16 molecules converted DCB and four did not (80% accuracy for the set of 20). This is consistent with the accuracy of the classification model and a significantly higher hit rate than observed in the initial survey, despite intentionally adding molecules that contained functional groups known to be reactive in this type of chemistry. Of the 16 molecules which converted DCB, 7 formed complex mixtures from which no single product could be isolated in appreciable yields. Notably, multiple different products could be isolated, and it is likely that with further condition optimization these could yield useful reactions; as such, these substrates could constitute interesting areas of future research.

**Figure 8:**
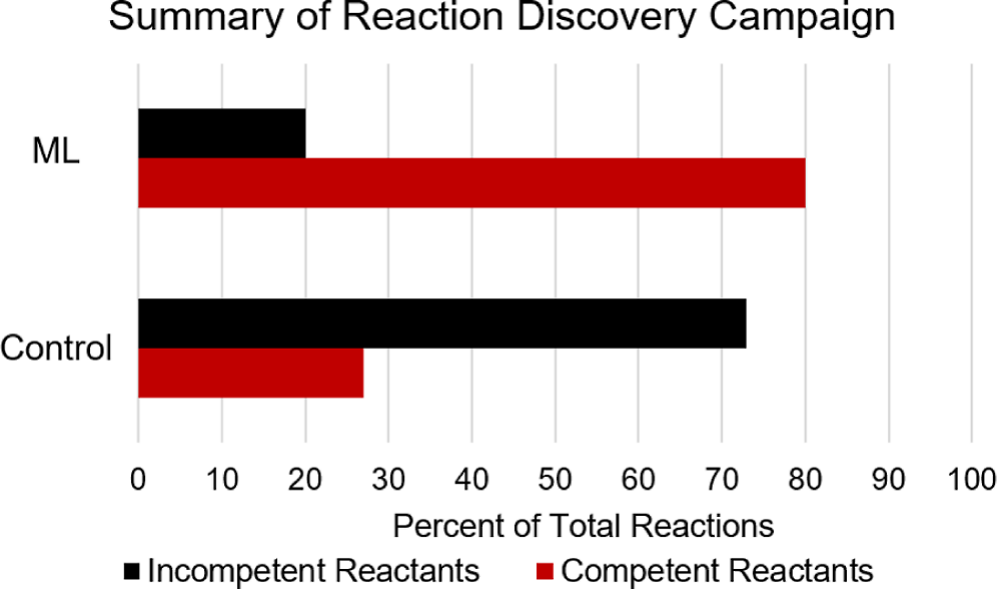
Summary of the reaction discovery campaign in percent. Top: molecules selected via the ML workflow; bottom: molecules from the initial survey. Molecules containing previously established functionality intentionally added to the training data have been removed for comparison.

The success of this experimental validation experiment should not be understated. As a comparison, 80% of the new reactions in the prediction set resulted in DCB conversion, whereas only 42% of the training reactions resulted in DCB conversion. This already drastic difference is compounded by the fact that 30 examples (21% of the total dataset) were known reactions intentionally added to the dataset that would not have been tested in a typical discovery campaign. If only unknown substrates are used as a comparison, only 27% of the training substrates convert DCB compared to the 80% from the prediction set (Figure 8). This substantial increase in hit rate is expected to dramatically improve the discovery rate in related screening campaigns. Further, in future implementations of this workflow, this data could be used to retrain the model for further improvements in domain applicability and prediction accuracy.

**Figure 9:**
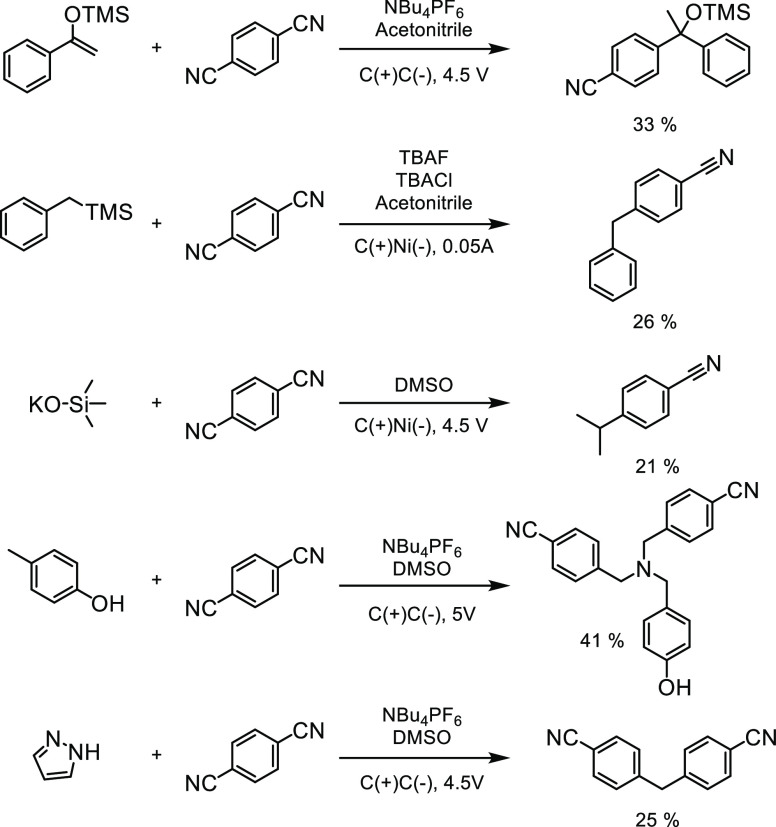
Selected newly discovered reaction predicted by the machine learning workflow and evaluated experimentally.

Of the remaining 9, some converted DCB but have limited synthetic utility. The sulfur and phosphorous-containing members of this set (Figure 7, labeled green) are simply oxidized and do not contain the DCB group, whereas the reaction with triethylsilane produced benzonitrile, albeit with a low conversion. Five of the reactions that convert DCB produced a major product that was isolable, and we considered them to be of either synthetic or mechanistic interest (Figure 9). Namely, the reaction of the silyl enol ether and the radical coupling of benzyltrimethylsilane with DCB are useful synthetic methods orthogonal to other means of constructing similar groups. For example, the TMS group is inert under many reaction conditions but can be electrochemically activated to arylate the benzylic position. From a discovery perspective, the remaining three examples are interesting as they constitute mechanistically ambiguous reactions that would not be rationally designed by an experimentalist. We believe that unveiling this reactivity will inspire mechanistic interrogation of these reactions, potentially unveiling new mechanistic regimes and enabling the rational design of new reactions using these insights. Additionally, these examples demonstrate the capability of this workflow to discover unintuitive transformations. As experimental throughput and analysis capabilities improve (or simply as more experiments are run), this capability will enable rapid exploration of reactivity space to rapidly develop new unique transformations. It is worth commenting on the low yields of the reported reactions; the goals of the study were to establish a workflow for the discovery of new reactions. As such, the optimization of the development of these reactions is beyond the scope of the current study. However, current work is underway to develop these reactions into synthetic methods, and we hope this work inspires other researchers to do the same.

## Conclusions

We have successfully (1) collected a suitable dataset of convergent paired electrolytic reactions for modeling endeavors, (2) developed a novel, general molecular representation containing DFT information which outperforms common 2D methods in multiple case studies, (3) developed a model capable of evaluating reactant candidates as competent or incompetent in an emerging field of chemistry, and (4) used this data to evaluate many reactive partners, yielding a high hit rate for newly discovered reactions. The molecular representation developed is particularly interesting, with apparent broader applications than reaction discovery. Also of note is the accuracy of the classification model used to evaluate reactant candidates. We believe this work to be foundational to data-guided discovery efforts. If the accuracy of this model is maintained, as it was in the current study, the rate-limiting step of method development would shift from reaction discovery to reaction optimization and development into a full synthetic method. This has the potential to make the development of new methodology significantly faster than current approaches, thus rapidly streamlining the development of emerging areas of chemistry. We look forward to broadening this workflow to include multiple reaction partners and reaction conditions in the analysis. Additional current efforts are underway to predict the overall transformation, increase experimental throughput, and streamline analysis. Additionally, we seek to couple this workflow with databases of commercially available molecules to automate the selection of new reactive mixtures.

## Supporting Information Available

General experimental, characterization data, spectra, and computational methods (PDF)
Datasets (ZIP)
Structure files (ZIP)
Example python code (ZIP)
