# Supplementary material for: Machine-Learning-Guided Discovery of Electrochemical Reactions
Source: J Am Chem Soc. 2022 Dec 2;144(49):22599–610. doi: 10.1021/jacs.2c08997 (PMC9756344; doi:10.1021/jacs.2c08997)
Supplement: Supplementary file 1 — ja2c08997_si_001.pdf [file ja2c08997_si_001.pdf]

Machine Learning-Guided Discovery of Electrochemical Reactions

Andrew F. Zahrt, Yiming Mo, Kakasaheb Y. Nandiwale, Ron Shprints, Esther Heid, Klavs F. Jensen*

Department of Chemical Engineering, Massachusetts Institute of Technology, Cambridge, MA, 02142

SUPPLEMENTARY INFORMATION

Table of Contents *Page*

General Information *S2*
Commercial Chemical Sources *S2*
Instrumentation *S2*
Droplet System Experiments *S4*
Scale Up Experiments *S11*
Experimental Protocols *S16*
Site Selectivity of Electrochemical Oxidation Case Study *S27*
Unsupervised Embedding Process and Case Studies *S31*
Large Scale DFT Dataset Calculation and Reactivity Prediction *S38*
NMR Data *S41*
References *S49*

General Information

Reactions were conducted under an atmosphere of dry nitrogen or argon which was passed through a drying tube equipped with calcium sulfate unless otherwise stated. Solvents used for extraction were reagent grade, and chromatography solvents were technical grade. Solvents used for reactions were HPLC grade and were stored over either 3 or 4 Ǻ molecular sieves. Normal phase column chromatography was performed using high-purity silica gel from Millipore Sigma (pore size = 60 Ǻ, 230-400 mesh particle size, 40-63 micron particle size) or RediSep Rf Gold Silica Gel columns (40 g) from Teledyne ISCO. Reverse phase chromatography was performed using Supelco C18-reversed phase silico gel. Retention factors, R_f, are reported for analytical thin layer chromatography performed on Baker-Flex silica gel IB-F sheet (5x20cm) with indicator purchased from VWR. Visualizations were accomplished by UV light or aqueous $KMnO_4$.

Commercial Chemical Sources

1,4-Dicyanobenzene (DCB), pyrazole, p-cresol, potassium trimethylsilanolate (90 %), 9-phenyl carbazole, furfuryl alcohol, phenyl hydrazine, acetonitrile, DMSO, thioanisole, dimethylphosphine, *tert*-butyl methyl sulfide, and thiobenzoic acid were ordered from Millipore Sigma and used without further purification. Workup and chromatography solvents were also purchased from Millipore Sigma and used as delivered. All electrolytes were ordered from Millipore Sigma and were high purity for electrolysis. Additionally, the smallest size was purchased to avoid water contamination from being open to atmosphere for long periods of time; opened bottles were stored in a jar with Drierite. Trimethylbenzylsilane was ordered from TCI and used without further purification. Methyl benzyl ether was ordered from Alfa Aesar and used without further purification. All glassy carbon was type two glassy carbon ordered from Alfa Aesar.

Instrumentation

^{1}H, and ^{13}C NMR spectra were recorded on Bruker Avance-III HD Nanobay 400

spectrometers, or a Bruker Avance Neo 500 spectrometer. Spectra are referenced to chloroform (δ = 7.26 ppm, ^{1}H; 77.0 ppm, ^{13}C). Chemical shifts are reported in ppm, and multiplicities are indicated by s (singlet), d (doublet), t (triplet), q (quartet), p (pentet), h (hextet), m (multiplet) and br (broad). Coupling constants, *J*, are reported in Hertz, and integration is provided and assignments are indicated. Mass spectrometry was performed in the MIT Department of Chemistry Instrumentation Facility. High resolution mass spectrometric data was collected using a JEOL AccuTOF 4G LC-plus equipped with an ionSense DART (Direct Analysis in Real Time) source. High resolution data are reported as calculated and measured masses to 10^{-4} mass accuracy.

Droplet System Experiments

A full description of the droplet system used for automated experimentation can is available in a previous publication.[1] In short, an automated liquid sampler (ALS) is used to prepare reaction droplets. The ALS prepares the droplet from stock solutions in vials on the reagent tray, then injects the droplets between two gas bubbles into a solvent stream. In this case, the ALS is housed inside a Plexiglas box that has been purged with nitrogen, and the inert gas surrounding the droplet is thus also nitrogen. A syringe pump pushes the solvent stream to a 6-way valve with a sample loop. On the inlet and outlet to the 6-way valve are phase sensors; when these phase sensors both detect gas, the droplet is in the sample loops, which triggers the valve to switch positions, connecting it to the reactor. The droplet is moved to the reactor by a syringe pump filled with argon gas. Once it reaches the reactor (as detected by a phase sensor which detects the location of the droplet) the reaction is started by the application of electricity and the droplet is oscillated in the reactor via a syringe pump with a syringe filled with argon. The reactor itself contains a platinum interdigitated electrode (IDE) with 5 µm spacing; when oscillating, the droplet is being passed along the surface of this IDE. Once the reaction is complete (as designated in this case by running for 20 minutes), the droplet is passed to another 6-way valve with a 5 µL sample loop. A phase sensor directly preceding the sample loop triggers the initiation of a

Chemstation method, which flips the 6-way valves and triggers the automated analysis.

In the course of this study, the reaction conditions were kept constant while the anodic reactive partner was varied. After testing a series of reactions previously tested in our laboratories,[2] a set of conditions which qualitatively gave conversion for each of the substrates were identified and used in all reactions run on the platform. Additionally, 4,4'-*tert*-butyl biphenyl was identified as an adequate internal standard, as it typically had a much longer retention time using reverse phase HPLC than any other analyte encountered in the study. Notably, because the identity of the products formed is unknown and many reactants have unknown retention times, it was not always possible to resolve the DCB peak. As such, conversion was not always able to be quantified due to overlapping peaks. However, in such cases it was clear by manually comparing the HPLC trace of the reaction without applying electricity with the HPLC trace of the reaction after electrolysis whether or not new species were formed. Owing to the nature of this analysis, the output was recorded like a Boolean variable – either conversion (1) or no conversion (-1). This was indeed adequate for target modeling task, which was designed to accommodate this experimental protocol. The preliminary results of this work are given in Table S1.

Table S1. Boolean outcomes of initial screening results.

Molecule	SMILES	Conversion?
3-acetyl pyridine	CC(C1=CN=CC=C1)=O	-1
triphenylphosphine	P(C1=CC=CC=C1)(C2=CC=CC=C2)C3=CC=CC=C3	-1
4(phenylsulfonyl)-benzonirile	N#CC1=CC=C(S(C2=CC=CC=C2)(=O)=O)C=C1	-1
1-(tert-butoxycarbonyl)piperidine-3-carboxylic acid	OC(C1CN(C(OC(C)(C)C)=O)CCC1)=O	-1
2,5-dichloroanisole	COC1=CC(Cl)=CC=C1Cl	-1
4-oxopentanoic acid	CC(CCC(O)=O)=O	-1

cyclopentanecarboxylic acid	OC(C1CCCC1)=O	-1
2,2-diphenylacetonitrile	N#CC(C1=CC=CC=C1)C2=CC=CC=C2	-1
3,3,3-trifluoropropanoic acid	FC(F)(F)CC(O)=O	-1
chlorocyclohexane	ClC1CCCCC1	-1
1-(methylsulfonyl)-1H-imidazole	CS(N1C=CN=C1)(=O)=O	-1
2-(hex-5-en-1-yl)oxirane	C=CCCCCC1CO1	-1
2-cyclopropylacetic acid	O=C(O)CC1CC1	-1
cyclobutanecarboxylic acid	OC(C1CCC1)=O	-1
1-methylcyclohexan-1-ol	CC1(O)CCCCC1	-1
cylcohexan-1-ol	OC1CCCCC1	-1
TBA cyanoborohydride	CCCC[N+](CCCC)(CCCC)CCCC.[BH3-]C#N	-1
Heptane	CCCCCCC	-1
Cyclohexane	C1CCCCC1	-1
Decane	CCCCCCCCCC	-1
Acetic Acid	CC(O)=O	-1
Trifluoroacetic acid	OC(C(F)(F)F)=O	-1
Benzene	C1=CC=CC=C1	-1
Biphenyl	C1(C2=CC=CC=C2)=CC=CC=C1	-1
pentanenitrile	CCCCC#N	-1
hexane	CCCCCC	-1

dichloromethane	ClCCl	-1
4-acetyl pyridine	O=C(C1=CC=NC=C1)C	-1
trifluorotoluene	FC(F)(F)C1=CC=CC=C1	-1
chlorobenzene	ClC1=CC=CC=C1	-1
fluorobenzene	FC1=CC=CC=C1	-1
2-chloropropane	CC(Cl)C	-1
1,2-dichloroethane	ClCCCl	-1
methyl nicotinate	O=C(OC)C1=CC=CN=C1	-1
1-(trifluoromethyl)cyclopropane-1-carboxylic acid	O=C(O)C1(C(F)(F)F)CC1	-1
(E)-3-methyloct-6-enoic acid	OC(CC(C)CC/C=C/C)=O	-1
1,2-dichlorobenzene	ClC1=C(Cl)C=CC=C1	-1
1-(tert-butyl)-4-fluorobenzene	CC(C)(C)C1=CC=C(F)C=C1	-1
1-(tert-butyl)-4-chlorobenzene	CC(C)(C)C1=CC=C(Cl)C=C1	-1
1-(tert-butyl)-4-(trifluoromethyl)benzene	CC(C)(C)C1=CC=C(C(F)(F)F)C=C1	-1
2-ethylhexanoic acid	CCCCC(CC)C(O)=O	-1
dodecane	CCCCCCCCCCCC	-1
4(3,5-dichlorophenoxy)butanoic acid	O=C(O)CCCOC1=CC(Cl)=CC(Cl)=C1	-1
diethyl ether	CCOCC	-1
propionic acid	CCC(O)=O	-1
hexanoic acid	CCCCCC(O)=O	-1

butanol	CCCO	-1
4-chloro pyridine	ClC1=CC=NC=C1	-1
glucose	O=C[C@@H]([C@H]([C@@H]([C@@H](CO)O)O)O)O	-1
triphenylphosphine oxide	O=P(C1=CC=CC=C1)(C2=CC=CC=C2)C3=CC=CC=C3	-1
1-hexyne	C#CCCCC	-1
DMA	CC(N(C)C)=O	-1
toluene	CC1=CC=CC=C1	-1
hydrocynamic acid	OC(CCC1=CC=CC=C1)=O	-1
sulfolane	O=S1(CCCC1)=O	-1
1-hexanol	CCCCCCO	-1
methanol	CO	-1
ethanol	CCO	-1
neopentyl alcohol	CC(C)(C)CO	-1
ethylene glycol	OCCO	-1
nitromethane	C[N+]([O-])=O	-1
CBr4	BrC(Br)(Br)Br	-1
isoquinoline	C12=CC=CC=C1C=CN=C2	-1
1-cyclohex-2-eneol (allylic alcohol)	OC1C=CCCC1	-1
3-ethylpyridine	CCC1=CN=CC=C1	-1
nitrosobenzene	O=NC1=CC=CC=C1	-1
3-pyridyl boronic acid	OB(O)C1=CN=CC=C1	-1

n-boc-pyrrolidine	O=C(N1CCCC1)OC(C)(C)C	-1
benzoic acid	O=C(O)C1=CC=CC=C1	-1
pivalic acid	CC(C)(C)C(O)=O	-1
tetrahydrofuran-2-carboxylic acid	O=C(O)C1CCCO1	-1
3-phenylpropan-1-ol	OCCCC1=CC=CC=C1	-1
1-bromo-4-ethylbenzene	BrC1=CC=C(CC)C=C1	-1
10-undecyne-1-ol	C#CCCCCCCCCCO	-1
4-methoxybenzoic acid	OC(C1=CC=C(OC)C=C1)=O	-1
tributylphoshine oxide	CCCCP(CCCC)(CCCC)=O	-1
n-methylpyrrolidine	CN1CCCC1	-1
n-benzylpiperidine	N1(CC2=CC=CC=C2)CCCCC1	-1
cyclohexene	C1=CCCCC1	-1
triethyl amine	CCN(CC)CC	-1
dibutyl phosphate	O=P(OCCCC)(O)OCCCC	-1
4-methyl(N,Ndimethyl)aniline	CC1=CC=C(N(C)C)C=C1	1
n-phenyl morpholine	C1(N2CCOCC2)=CC=CC=C1	1
1-cyclohex-2-eneol (allylic alcohol) - PMP carbamate	O=C(NC1=CC=C(OC)C=C1)OC2C=CCCC2	1
1,3-dioxoisoindolin-2-yl 4-phenylbutanoate	O=C(ON1C(C(C=CC=C2)=C2C1=O)=O)CCCC3=CC=CC=C3	1
1-tbu-1-cyclohexene	CC(C)(C)C1=CCCCC1	1
3-butenoic acid	C=CCC(O)=O	1

1,3-dioxoisoindolin-2-yl 4-((tert-butoxycarbonyl)amino)cyclohexane-1-carboxylate	O=C(OC(C)(C)C)NC1CCC(C(ON2C(C(C=CC=C3)=C3C2=O)=O)=O)CC1	1
n-boc-piperidine	O=C(N1CCCCC1)OC(C)(C)C	1
1,3-dioxoisoindolin-2-yl (tert-butoxycarbonyl)-L-phenylalaninate	O=C(ON1C(C(C=CC=C2)=C2C1=O)=O)[C@H](CC3=CC=CC=C3)NC(OC(C)(C)C)=O	1
1,3-dioxoisoindolin-2-yl acetyl-L-phenylalaninate	O=C(ON1C(C(C=CC=C2)=C2C1=O)=O)[C@H](CC3=CC=CC=C3)NC(C)=O	1
tetrabutylammonium benzoylalaninate	CC(NC(C1=CC=CC=C1)=O)C([O-])=O.CCCC[N+](CCCC)(CCCC)CCCC	1
2-(4-methoxyphenyl)acetic acid	O=C(O[H])CC1=CC=C(OC)C=C1	1
1-allyl-4-methoxybenzene	C=CCC1=CC=C(OC)C=C1	1
dehydrocholic acid	O=C(CC1CC([C@@]2([H])[C@]3([H])CC[C@@H]([C@]34C)[C@H](C)CCC(O)=O)=O)CC[C@]1(C)[C@@]2([H])CC4=O	1
Ethylidenecyclohexane	C/C=C1CCCCC/1	1
2,4,4-trimethylpent-1-ene	C=C(C)CC(C)(C)C	1
furfuryl alcohol	OCC1=CC=CO1	1
dabco	N1(CC2)CCN2CC1	1
2-(4-methoxy-3-(methoxycarbonyl)phenyl)acetic acid	O=C(OC)C1=C(OC)C=CC(CC(O)=O)=C1	1
indometacine	CC1=C(C2=C(N1C(C3=CC=C(Cl)C=C3)=O)C=CC(OC)=C2)CC(O)=O	1
ascorbic acid	OC([C@H](O1)[C@H](CO)O)=C(O)C1=O	1
tartaric acid	O[C@@H]([C@@H](C(O)=O)O)C(O)=O	1
ibuprphen	CC(CC1=CC=C(C(C(O)=O)C)C=C1)C	1

1-phenyl pyrrolidine	N1(C2=CC=CC=C2)CCCC1	1
n,n-diethylaniline	CCN(CC)C1=CC=CC=C1	1
3-methylcyclohex-2-en-1-one	O=C1C=C(C)CCC1	1
benzyl alcohol	OCC1=CC=CC=C1	1
sodium benzenesulfinate	O=S(O[Na])C1=CC=CC=C1	1
tatrabutylammonium 4-methylbenzenesulfinate	CCCC[N+](CCCC)(CCCC)CCCC.O=S(C1=CC=C(C)C=C1)[O-]	1
methylene blue	CN(C)C1=CC2=C(C=C1)N=C3C(C=C(C=C3)N(C)C)=[S+]2.[Cl-]	1
CDI	O=C(N1C=NC=C1)N2C=NC=C2	1
N-hydroxyphthalamide	O=C(N1O)C2=CC=CC=C2C1=O	1
N-phenyl carbazole	N1(C2=CC=CC=C2)C3=C(C4=C1C=CC=C4)C=CC=C3	1
4-ethyl anisole	CCC1=CC=C(OC)C=C1	1
azobenzene	C1(/N=N/C2=CC=CC=C2)=CC=CC=C1	1
4-methyl quinoline	CC1=CC=NC2=CC=CC=C21	1
1-methyl-1H-benzo[d]imidazole	CN1C=NC2=C1C=CC=C2	1
benzoxazole	C1(N=CO2)=C2C=CC=C1	1
1-me-1,2,4-triazole	CN1N=CN=C1	1
2-benzyl-1H-benzo[d]imidazole	C12=CC=CC=C1NC(CC3=CC=CC=C3)=N2	1
(methoxymethyl)benzene	COCC1=CC=CC=C1	1
benzothiazole	C12=CC=CC=C1SC=N2	1
benzyl bromide	BrCC1=CC=CC=C1	1
2,4,6-trimethoxypyridine	COC1=CC(OC)=CC(OC)=N1	1

phenyl hydrazine	NNC1=CC=CC=C1	1
dimethyl ethylidene malonate	O=C(OC1C)C(C)(C)C(O1)=O	1
methyl (E)-but-2-enoate	C/C=C/C(OC)=O	1
10H-phenothiazine	C12=CC=CC=C1NC3=C(C=CC=C3)S2	1
3-me indole	CC1=CNC2=C1C=CC=C2	1
2-benzylbenzimidazole	C1(CC2=CC=CC=C2)=NC3=CC=CC=C3N1	1
pyrene	C12=CC=C3C=CC=C4C=CC(C2=C34)=CC=C1	1
thymolphthalein	O=C1OC(C2=CC(C)=C(O)C=C2C(C)C)(C3=CC(C)=C(O)C=C3C(C)C)C4=C1C=CC=C4	1
Trimethoxybenzene	COC1=CC(OC)=CC(OC)=C1	1
benzil	O=C(C1=CC=CC=C1)C(C2=CC=CC=C2)=O	1
2-benzyl pyridine	C1(CC2=CC=CC=C2)=NC=CC=C1	1
ethyl benzene	CCC1=CC=CC=C1	1
THF	C1CCOC1	1
2,4,6-trimethyl aniline	NC1=C(C)C=C(C)C=C1C	1
1,2,3,4-tetrahydronaphthan-1-ol	OC1CCCC2=CC=CC=C21	1
Na2S	NaSNa	1

Scale Up Experiments

Although the droplet system provided a method by which to rapidly generate an initial dataset rapidly with small quantities of material, the quantity of product produced was too small to thoroughly analyze the reaction mixture. Consequently, we required a system in which it was possible to achieve similar reactivity as observed using the droplet system but operate on larger

scales in order to facilitate isolation and full characterization of new reaction components. To achieve this, we employed a recirculating setup similar to that previously used in or laboratories.[2] In this setup, parallel plate reactors were employed which either contained 5cm x 5cm electrodes (made of either glassy carbon or nickel) or 10cm x 10cm glassy carbon plates. Full designs for both reactor types are available in reference **Error! Bookmark not defined.**. The reactors were attached either to a DC power supply for reactions that did not require switching polarity or to the power supply on the droplet system, which was controlled manually to set the voltage and switch time. Vici Valco positive displacement pumps were used to pump the reaction mixture from the reaction vessel (a culture tube), through the reactor, and back into the reaction vessel. The vessels contained a stir bar and were placed over a stir plate for mixing. The pumps were controlled with labview code which has been published previously by our laboratory.[3] An image of the reactions is shown in Figure S1 (argon balloons in culture tubes have been removed for clarity).

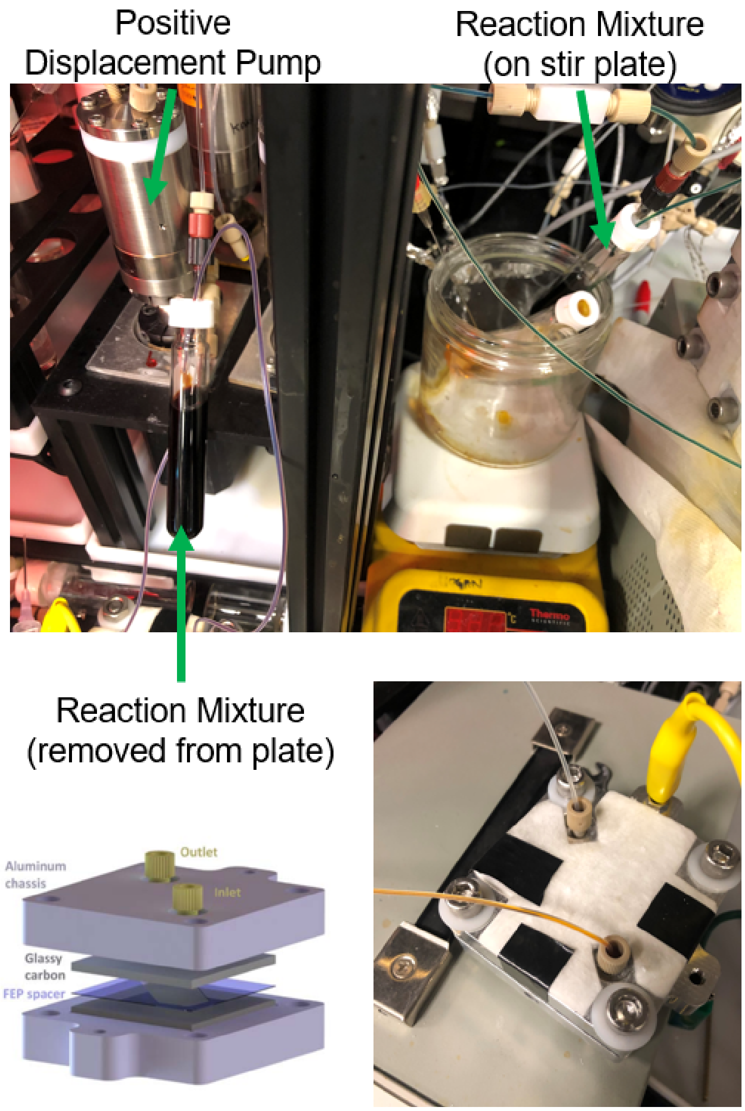

Figure S1. Recirculating reaction setup and diagram of parallel plate reactor.

Although this reactor setup was able to recapitulate the reactivity observed on the droplet system in some cases, in others it was less successful. Using this setup, a subset of reactants of tested from each category above. A qualitative summary of results is provided in Table 1.

Table S2. Examination of Selected New Reactants

Molecule	Category	Scale Up (Recirculating)
Na2S	Misc.	Complex Mixture
Acrylonitrile	Misc.	Complex Mixture
phenyl hydrazine	Misc.	Competent Reaction
benzyl bromide	Misc.	Complex Mixture

Trimethoxybenzene	AR	No Reaction
N-phenyl carbazole	AR	No Reaction
Benzoxazole	AR	No Reaction
Benzothiazole	AR	No Reaction
Pyrene	AR	No Reaction
furfural alcohol	BN	Competent Reaction
benzyl methyl ether	BN	Competent Reaction
THF	CH	Complex Mixture

Even after extensive testing, half of the reactions demonstrated to convert 1,4-dicyanobenzene on the droplet system did not convert using the recirculating reactor system. Notably, all of these reactants contain an electron rich π-system or extended π-surface. Interestingly, these reaction mixtures all exhibited a very high current at constant voltage, but even after passing excessive quantities of current no conversion was observed. In these cases, we reasoned that some electron shuttle mechanism may be in play (e.g., DCB forms a stable radical anion, other reactant forms stable radical cation, an electron is exchanged to reform the starting material). A possible explanation for the difference in reactivity is the electrode material. To investigate, we tested a batch reaction using platinum mesh electrodes with switching polarity with 9-phenylcarbazole as the reactant in DMSO with tetrabutylammonium hexafluorophosphate as the supporting electrolyte. The reaction was run at constant current of 3 V and the polarity was switched every 20 seconds. In this case, the dimerization of 9-phenylcarbazole was clearly observed. Notably, as the reaction progressed, a dark brown solid formed on the electrode surface, requiring the electrodes to be cleaned 3 times over the course of the electrolysis. The electrolysis was stopped after 1 hr of run time, and the crude NMR clearly showed three species: 9-phenylcarbazole, 9-phenylcarbazole dimer, and DCB. The mixture was purified via silica gel chromatography using 5 % ethyl acetate / 95 % hexanes mixture to isolate and characterize the dimer (a white solid). The 1H-NMR data for the dimer was found to be in line with previous reports.[4]

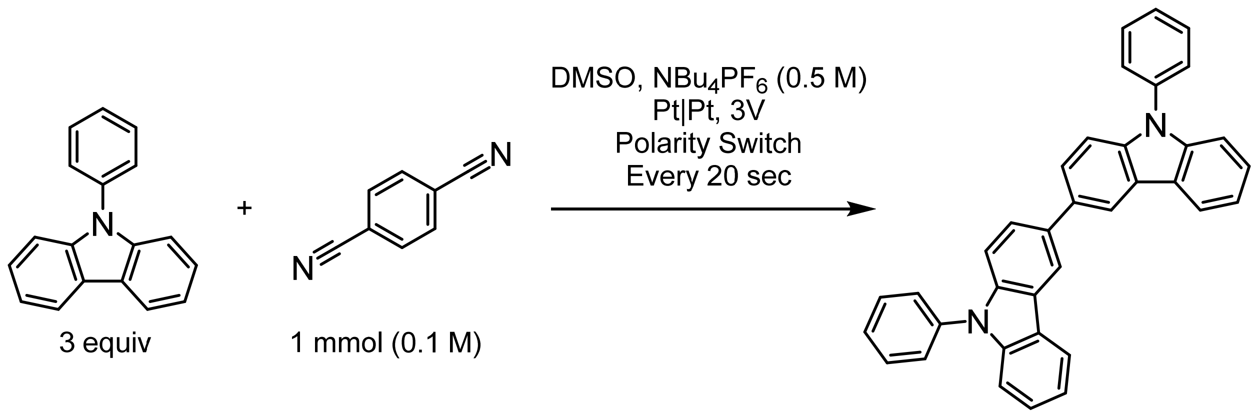

Having been able to reproduce the reactivity observed on the droplet system, a few reactions from the original training set were selected for further interrogation. Notably, to facilitate purification and characterization, some rough optimization was performed to simplify the reaction mixtures. This process was taken in general to convert reactions discovered on the droplet system to the recirculating platform. If other researchers are interested in using the predicted outcomes in the supplemental files to explore new reactions, we have found this general routine was typically adequate to get a good lead set of reaction conditions:

(1) The reactions were run on scale using identical conditions to the droplet system

(2) If this mixture was either complex or no significant conversion was observed, either pure DMSO or acetonitrile was used with no base and tetrabutylammonium hexafluorophosphate as supporting electrolyte. For selecting a voltage, the reactions were run at constant potential and the voltage was gradually increase up to 5 V until DCB began to convert.

(3) If this failed (typically owing to the mixture being complex, meaning that no one new species was formed predominately), a base was added (either LiOAc for DMSO or lutidine for acetonitrile or DMSO).

(4) If this failed, the process was repeated but with a nickel cathode rather than a glassy carbon cathode.

(5) For some reactions, some products were able to be identified but the mixture still too complex. In these cases, a literature search was done to identify similar reactions. If similar reactions exist, the conditions from these were tested. If not, the mixture was simply labeled "complex mixture"

(6) If no one new component of the mixture was produce as a major product (again, meaning that one new species is formed in significantly greater quantities than other new species as determined by the crude proton NMRs), the mixture was labeled as a complex mixture. Notably, some species were able to be identified and it is conceivable that some reactions labeled as complex might be optimizable. This investigation is outside the scope of the current work, but constitutes a future direction in which reaction conditions are added to the virtual screen rather than just reactants.

Experimental Protocols for Exemplar Reactions

Procedure for analysis of thioanisole, tert-butylmethylsulfide, dimethylphenylphosphine, and triethylsilane reactions.

With the general protocol for converting new droplet system reactions to scaled up reactions developed, reactions discovered during the collection of training data and analysis of the predicted set of reactive compounds were scaled up for characterization. Referring to Figure 6 in the main text, the compounds in green were tested under identical reaction conditions. In these cases, DCB (0.5 mmol, 64 mg) was added to a 20 mL scintillation vial. To this vessel was added NBu_4PF_6 (1 mmol, 387 mg), 5 mL solvent (2:1 DMSO:Acetonitrile, both degassed via sparging with argon separately), 2,6-lutidine (2.5 mmol, 290 µL), 5 equiv of the other reactant, and lithium acetate (1 mmol, 66 mg). This mixture was stirred until all reaction components dissolved (~45 min). Meanwhile, a culture tube was fitted with a magnetic stir bar and septum cap and purged with argon. After purging, the vent needle was replaced with a needle leading to a balloon, which was then filled and emptied 3x. After the final purge, the filled balloon was left to ensure the culture tube remained under positive pressure argon. The solution prepared in the

scintillation vial was then taken up in a syringe which was then fitted with a 0.2 µm PTFE filter with a needle on the outlet and filtered into the culture tube. This culture tube was then transferred to the recirculating flow setup and clamped above a magnetic stir plate. Two units of FEP tubing were inserted into the vial – the inlet and outlet to the flow path. The inlet led to a Vici Valco positive displacement pump (previously described), which then lead to the large echem parallel plate reactor with 10 cm x 10 cm glassy carbon plates. The reaction was circulated through the system, and electricity was applied. The reactions were run at 3.5 V constant potential, switching polarity every 20 seconds. After 5 hr of continuous run time, the reactions were stopped. The reactions were then poured into a 60 mL separatory funnel, to which 20 mL of water was added. This mixture was extracted 3 times with 10 mL dichloromethane, with the organic layers dried over magnesium sulfate. The combined organics were filtered, the drying agent rinsed with 20 mL dichloromethane, after which the solvent was removed by rotary evaporation. The reactions were then analyzed by crude NMR. For thioanisole and *tert*-butyl methyl sulfide, the corresponding sulfoxides were formed.[5,6] Similarly, dimethylphenylphosphine was oxidized to the corresponding phosphine oxide.[7] In the case of triethylsilane, low conversion was observed along with small quantities of benzonitrile. Notably, triethylsilane was not miscible with the reaction solvent; in fact, if the reaction was not stirred and the inlet to the recirculating setup placed at the bottom of the culture tube, no conversion was observed. We postulate that devising a reaction system in which all components are miscible could turn this into a viable reaction. However, the synthetic utility of other reactions seemed greater than for this reaction; as such, we decided to forgo additional experimentation for this reaction.

4-(methoxy(phenyl)methyl)benzonitrile

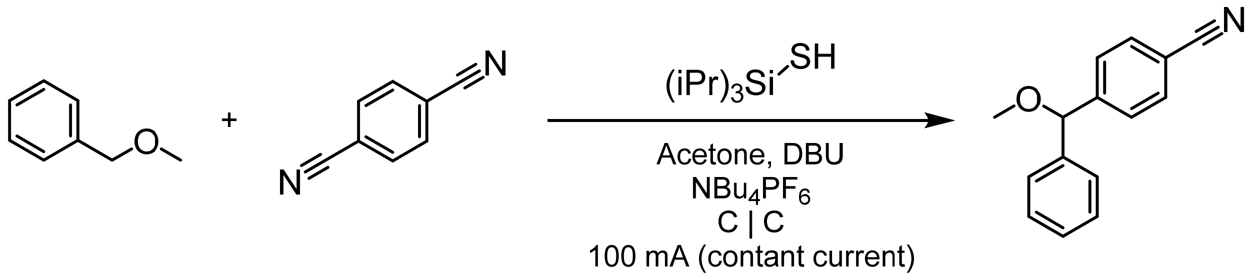

To a 20 mL scintillation vial was added DCB (1 mmol, 128 mg). To this vessel was added methyl benzyl ether (10 mmol, 1.24 mL), tetrabutylammonium hexafluorophosphate (2 mmol, 774 mg), DBU (0.4 mmol, 60 µL) and 5 mL acetone (degassed via sparging with argon). This mixture was stirred until all reaction components dissolved (~10 min). Meanwhile, a second scintillation vial was charged with triisopropylsilanethiol (0.5 mmol, 215 µL) and 5 mL of acetone. The solutions were loaded into two separate 10 mL stainless steel syringes. These outlets were joined via a T-connection, which was fed into the large echem parallel plate reactor with 10 cm x 10 glassy carbon plates. Both syringes were placed on a Harvard PhD syringe pump and the mixtures pumped at 3 µL/min. The reactions were run at 3.25 V constant voltage, switching polarity every 20 sec. The first 0.5 mL of reaction mixture exiting the reactor was discarded, and the next 2.5 mL collected. This 2.55 mL was added to a 60 mL separatory funnel, to which 20 mL of water was added. This mixture was extracted 3 times with 10 mL dichloromethane, with the organic layers dried over magnesium sulfate. The combined organics were filtered, the residual drying agent rinsed with 20 mL dichloromethane, after which the solvent was removed by rotary evaporation. This crude mixture was deposited silica gel and dry loaded onto a column (20 cm diameter). The mixture was purified by silica gel chromatography using 5 % ethyl acetate in hexanes, isolating 71 mg (32 %) of the desired compound. The 1H and 13C data matched what has been previously reported.[8]

^{1}H NMR (500 MHz, $CDCl_3$) δ 7.62 (d, J = 8.3 Hz, 2H), 7.48 (d, J = 8.3 Hz, 2H), 7.37–7.30 (m, 5H),

5.27 (s, 1H), 3.39 (s, 3H)

^{13}C NMR (125 MHz, $CDCl_3$) δ 147.58, 140. 60, 132.24, 128.71, 128.12, 127.26, 126.99, 84.58, 57.08

4-(furan-2-ylmethyl)benzonitrile

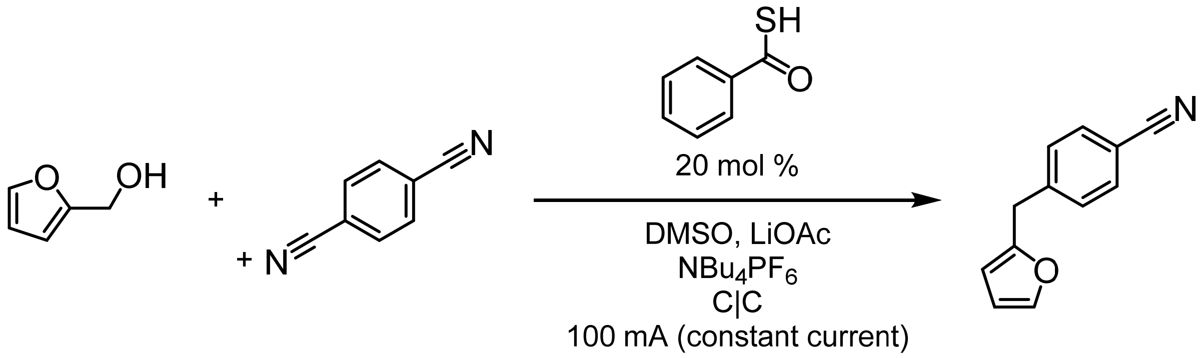

To a 20 mL scintillation vial was added DCB (0.5 mmol, 64 mg). To this vessel was added NBu_4PF_6 (1 mmol, 387 mg), 5 mL DMSO (degassed via sparging with argon), lithium acetate (1 mmol, 66 mg), thiobenzoic acid (0.1 mmol, 14 mg), and furfuryl alcohol (5 mmol, 0.435 mL). This mixture was stirred until all reaction components dissolved (~45 min). Meanwhile, a culture tube was fitted with a magnetic stir bar and septum cap and purged with argon. After purging, the vent needle was replaced with a needle leading to a balloon, which was then filled and emptied 3x. After the final purge, the filled balloon was left to ensure the culture tube remained under positive pressure argon. The solution prepared in the scintillation vial was then taken up in a syringe which was then fitted with a 0.2 µm PTFE filter with a needle on the outlet and filtered into the culture tube. This culture tube was then transferred to the recirculating flow setup and clamped above a magnetic stir plate. Two units of FEP tubing were inserted into the vial – the inlet and outlet to the flow path. The inlet led to a Vici Valco positive displacement pump (previously described), which then lead to the large echem parallel plate reactor with 10 cm x 10 cm glassy carbon plates. The reaction was circulated through the system, and electricity was applied. The reactions were run at 100 mA constant current. After 5 hr of continuous run time, the reaction was stopped. The reactor setup was then flushed with an additional 30 mL DMSO. The reactions were then poured

into a 125 mL separatory funnel, to which 50 mL of water was added. This mixture was extracted 3 times with 20 mL dichloromethane, with the organic layers dried over magnesium sulfate. The combined organics were filtered, the residual drying agent rinsed with 30 mL dichloromethane, after which the solvent was removed by rotary evaporation. This crude mixture was deposited silica gel and dry loaded onto a column (20 cm diameter). The mixture was purified by silica gel chromatography using a gradient of pure hexanes, 1 % ethyl acetate in hexanes, and 2 % ethyl acetate in hexanes. The product was isolated as a colorless oil in 24 % yield (22 mg). Notably, the C-H functionalization product was also recovered in 20 % yield. The 1H and 13C data matched what has been previously reported.[9]

^{1}H NMR (400 MHz, $CDCl_3$) δ 7.59 (d, J = 8.3 Hz, 2H), 7.32 (d, J = 8.5 Hz, 3H), 6.31 (dd, J = 3.0, 1.9 Hz, 1H), 6.06 (dd, J = 3.1, 0.6 Hz, 1H), 4.03 (s, 2H) ppm

^{13}C NMR (100 MHz, $CDCl_3$) δ 152.6, 143.7, 142.0, 132.3, 129.4, 118.8, 110.6, 110.4, 107.0, 34.5

4-aminobenzonitrile

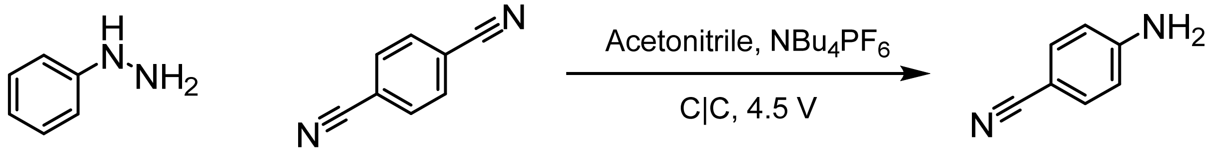

To a 20 mL scintillation vial was added DCB (0.5 mmol, 64 mg). To this vessel was added NBu_4PF_6 (1 mmol, 387 mg), 5 mL acetonitrile (degassed via sparging with argon), and phenyl hydrazine (5 mmol, 0.493 mL). This mixture was stirred until all reaction components dissolved (~45 min). Meanwhile, a culture tube was fitted with a magnetic stir bar and septum cap and purged with argon. After purging, the vent needle was replaced with a needle leading to a balloon, which was then filled and emptied 3x. After the final purge, the filled balloon was left to ensure the culture tube remained under positive pressure argon. The solution prepared in the scintillation vial was then taken up in a syringe which was then fitted with a 0.2 μm PTFE filter with a needle on the outlet and filtered into the culture tube. This culture tube was then transferred to the recirculating flow setup and clamped above a magnetic stir plate. Two units of FEP tubing were

inserted into the vial – the inlet and outlet to the flow path. The inlet led to a Vici Valco positive displacement pump (previously described), which then lead to the large echem parallel plate reactor with 10 cm x 10 cm glassy carbon plates. The reaction was circulated through the system, and electricity was applied. The reactions were run at 4.5 V, constant voltage, switching polarity every 20 sec. After 12 hr of continuous run time, the reaction was stopped. The reactor setup was then flushed with 30 mL DMSO. The reactions were then poured into a 125 mL separatory funnel, to which 50 mL of water was added. This mixture was extracted 3 times with 20 mL dichloromethane, with the organic layers dried over magnesium sulfate. The combined organics were filtered, the residual drying agent rinsed with 30 mL dichloromethane, after which the solvent was removed by rotary evaporation. This crude mixture was deposited silica gel and dry loaded onto a column (20 cm diameter). The mixture was purified by silica gel chromatography using 30 % ethyl acetate in hexanes. This was not sufficient to fully purify the product, so all fraction containing the desired product were combined and concentrated, and the product was then crystallized using DCM/Heptane mixture. The product was afforded as a yellow solid (23 mg, 40 %). The 1H and 13C data matched what has been previously reported.[10]

^{1}H NMR (400 MHz, $CDCl_3$): δ 7.41 (dt, 2H, J = 8.8 Hz), 6.64 (dt, 2H, J = 8.8), 4.15 (br s, 2H)

^{13}C NMR (100 MHz, $CDCl_3$): δ 150.18,133.66, 119.94, 114.28, 100.12

4-(1-phenyl-1-((trimethylsilyl)oxy)ethyl)benzonitrile

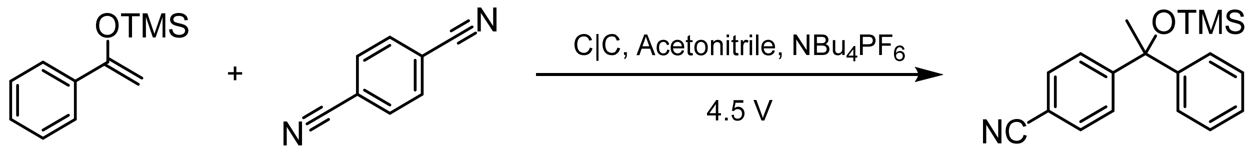

To a 20 mL scintillation vial was added DCB (0.5 mmol, 64 mg). To this vessel was added NBu_4PF_6 (1 mmol, 387 mg), 5 mL acetonitrile (degassed via sparging with argon), and trimethyl((1-phenylvinyl)oxy)silane (5 mmol, 962 mg). This mixture was stirred until all reaction components dissolved (~45 min). Meanwhile, a culture tube was fitted with a magnetic stir bar and septum cap and purged with argon. After purging, the vent needle was replaced with a needle

leading to a balloon, which was then filled and emptied 3x. After the final purge, the filled balloon was left to ensure the culture tube remained under positive pressure argon. The solution prepared in the scintillation vial was then taken up in a syringe which was then fitted with a 0.2 µm PTFE filter with a needle on the outlet and filtered into the culture tube. This culture tube was then transferred to the recirculating flow setup and clamped above a magnetic stir plate. Two units of FEP tubing were inserted into the vial – the inlet and outlet to the flow path. The inlet led to a Vici Valco positive displacement pump (previously described), which then lead to the large echem parallel plate reactor with 10 cm x 10 cm glassy carbon plates. The reaction was circulated through the system, and electricity was applied. The reactions were run at 4.5 V, constant voltage, switching polarity every 20 sec. After 2 hr of continuous run time, the reaction was stopped. The reactor setup was then flushed with 30 mL DMSO. The reactions were then poured into a 125 mL separatory funnel, to which 50 mL of water was added. This mixture was extracted 3 times with 20 mL dichloromethane, with the organic layers dried over magnesium sulfate. The combined organics were filtered, the residual drying agent rinsed with 30 mL dichloromethane, after which the solvent was removed by rotary evaporation. This crude mixture was deposited silica gel and dry loaded onto a column (20 cm diameter). The mixture was purified by silica gel chromatography using 10 % ethyl acetate in hexanes. This was not sufficient to fully purify the product, so all fractions containing the desired product were combined chromatographed again using a gradient from 0 % to 5 % ethyl acetate in hexanes. The product was collected as a colorless oil (49 mg, 33 %)

^{1}H NMR (400 MHz, $CDCl_3$): δ 7.56 (d, 2H, J = 8.8 Hz), 7.47 (d, 2H, J = 8.8 Hz), 7.23-7.34(m, 5H), 1.94 (s, 3H), -0.01 (s, 9H).

^{13}C NMR (101 MHz, $CDCl_3$) δ 155.17, 147.41, 131.81, 128.14, 127.21, 126.59, 126.26, 119.05, 110.23, 78.25, 30.16, 1.96.

HRMS: *n*+1 formula: C18H22NOSi. Calc: 296.14652. Measured: 296.1428.

4-isopropylbenzonitrile

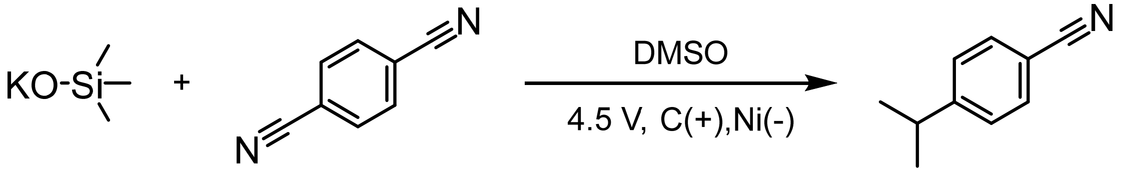

To a 20 mL scintillation vial was added DCB (0.5 mmol, 64 mg). To this vessel was added TMSOK (5 mmol, 641.45 mg), and 5 mL DMSO (degassed via sparging with argon). This mixture was stirred until all reaction components dissolved (~45 min). Meanwhile, a culture tube was fitted with a magnetic stir bar and septum cap and purged with argon. After purging, the vent needle was replaced with a needle leading to a balloon, which was then filled and emptied 3x. After the final purge, the filled balloon was left to ensure the culture tube remained under positive pressure argon. The solution prepared in the scintillation vial was then taken up in a syringe which was then fitted with a 0.2 µm PTFE filter with a needle on the outlet and filtered into the culture tube. This culture tube was then transferred to the recirculating flow setup and clamped above a magnetic stir plate. Two units of FEP tubing were inserted into the vial – the inlet and outlet to the flow path. The inlet led to a Vici Valco positive displacement pump (previously described), which then lead to the large echem parallel plate reactor with 5 cm x 10 5 glassy carbon plate (anode) and nickel (cathode) plates. The reaction was circulated through the system, and electricity was applied. The reactions were run at 4.5 V constant voltage. Until complete conversion of DCB as monitored by 1H NMR aliquots, after which the reaction was stopped. The reactor setup was then flushed with 30 mL DMSO. The reaction was then poured into a 125 mL separatory funnel, to which 50 mL of water was added. This mixture was extracted 3 times with 20 mL dichloromethane, with the organic layers dried over magnesium sulfate. The combined organics were filtered, the residual drying agent rinsed with 30 mL dichloromethane, after which the solvent was removed by rotary evaporation. This crude mixture was deposited silica gel and dry loaded onto a column (20 cm diameter). The mixture was purified by silica gel chromatography using 1 % ethyl acetate

in hexanes. The product was afforded as a colorless oil (15 mg, 21 %). The 1H and 13C data matched what has been previously reported.[11]

^{1}H NMR (500 MHz, $CDCl_3$) δ 7.58 (d, J = 8.4 Hz, 1H), 7.32 (d, J = 8.0 Hz, 1H), 2.96 (p, J = 6.9 Hz, 1H), 1.26 (d, J = 6.9 Hz, 5H).

^{13}C NMR (126 MHz, $CDCl_3$) δ 154.37, 132.25, 127.31, 119.20, 109.63, 34.41, 23.55.

4-Benzylbenzonitrile

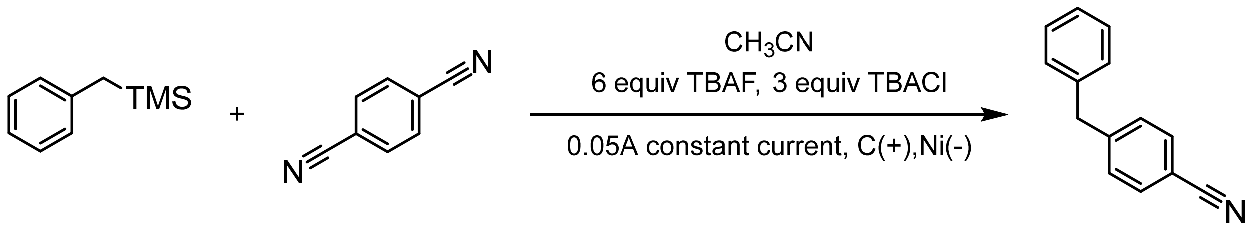

To a 20 mL scintillation vial was added DCB (1 mmol, 128 mg). To this vessel was added TMSBn (10 mmol, 1.64 g) and 10 mL DMSO (degassed via sparging with argon). This mixture was stirred until all reaction components dissolved (~45 min). Meanwhile, a second scintillation vial was charged with TBAF hydrate (6 mmol anhydrous basis, 1.45 g), TBACl (3 mmol, 834 mg), and 10 mL of DMSO. The solutions were loaded into two separate 10 mL stainless steel syringes. These outlets were joined via a T-connection, which was fed into the small echem parallel plate reactor with 5 cm x 5 glassy carbon plate (anode) and nickel (cathode) plates. Both syringes were placed on a Harvard PhD syringe pump and the mixtures pumped at 5µL/min. The reactions were run at 4.5 V constant voltage. The first 0.5 mL of reaction mixture exiting the reactor was discarded, and the next 5 mL collected. This 5 mL was added to a 60 mL separatory funnel, to which 20 mL of water was added. This mixture was extracted 3 times with 10 mL dichloromethane, with the organic layers dried over magnesium sulfate. The combined organics were filtered, the residual drying agent rinsed with 20 mL dichloromethane, after which the solvent was removed by rotary evaporation. This crude mixture was deposited silica gel and dry loaded onto a column (20 cm diameter). The mixture was purified by silica gel chromatography using 1 % ethyl acetate in hexanes. The product was afforded as a white solid (25 mg, 26 %). The 1H and 13C data

matched what has been previously reported.[12]

^{1}H NMR (400 MHz, $CDCl_3$) δ 7.56 (d, J = 8.3 Hz, 2H), 7.31 (m, 5H), 7.19 (d, J = 7.3 Hz, 2H), 4.04 (s, 2H)

^{13}C NMR (100 MHz, $CDCl_3$) δ 146.8, 139.4, 132.3 (2C), 129.7 (2C), 129.0 (2C), 128.8 (2C), 126.7, 119.1, 110.0, 42.0

4,4'-(((4-hydroxyphenyl)azanediyl)bis(methylene))dibenzonitrile

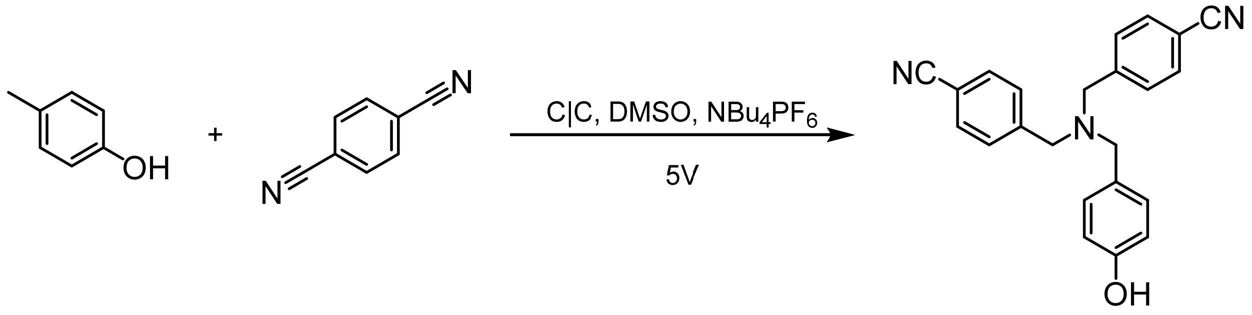

To a 20 mL scintillation vial was added DCB (1 mmol, 128 mg). To this vessel was added *p*-cresol (10 mmol, 1.084 g), NBu_4PF_6 (1 mmol, 387 mg), and 10 mL DMSO (degassed via sparging with argon). To this vessel was added a stir bar, and the mixture was sparged with argon while stirring for 30 min. Two classy carbon electrodes were added through the septum, and the mixture was heated to 70 °C. The mixture was electrolyzed at 5V constant potential for 12 hr. The reaction was then poured into a 125 mL separatory funnel, to which 50 mL of water was added. This mixture was extracted 3 times with 20 mL dichloromethane, with the organic layers dried over magnesium sulfate. The combined organics were filtered, the residual drying agent rinsed with 30 mL dichloromethane, after which the solvent was removed by rotary evaporation. This crude mixture was deposited silica gel and dry loaded onto a column (20 cm diameter). The mixture was chromatographed using a gradient from 20 % dichloromethane in hexanes to pure dichloromethane. This was not sufficient to purify the mixture, so all fractions containing the desired product were combined and subjected to reverse phase chromatography using a gradient from 25 % acetonitrile in water to pure acetonitrile. The product was afforded as a pale-yellow

solid (72 mg, 41 %).

^{1}H NMR (400 MHz, $CDCl_3$) δ 7.62 (d, J = 8.3 Hz, 4H), 7.60 (d, J = 8.3 Hz, 4H), 7.47 (d, J = 8.6 Hz, 2H), 7.46 (d, J = 8.6 Hz, 2H), 7.19 (d, J = 8.3 Hz, 2H), 7.17 (d, J = 8.3 Hz, 2H), 6.81 (d, J = 8.3 Hz, 2H), 6.78 (d, J = 8.3 Hz, 2H), 3.57 (s, 4H), 3.46 (s, 2H).

^{13}C NMR (101 MHz, $CDCl_3$) δ 154.95, 144.98, 132.26, 130.10, 129.19, 118.84, 115.26, 111.03, 57.69, 57.65.

HRMS: *n*+1 formula: C23H19N3O. Calc: 354.16009. Measured: 354.15887.

4,4'-methylenedibenzonitrile

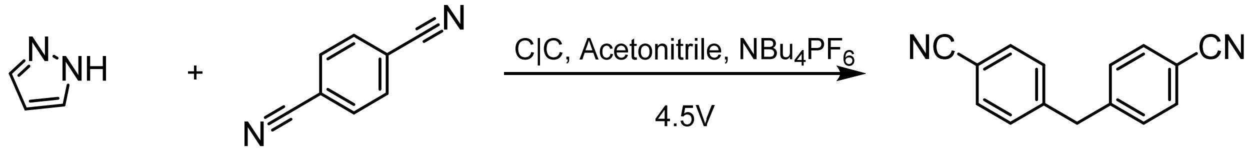

To a 20 mL scintillation vial was added DCB (0.5 mmol, 64 mg). To this vessel was added NBu_4PF_6 (1 mmol, 387 mg), 5 mL acetonitrile (degassed via sparging with argon), and pyrazole (5 mmol, 340 mg). This mixture was stirred until all reaction components dissolved (~45 min). Meanwhile, a culture tube was fitted with a magnetic stir bar and septum cap and purged with argon. After purging, the vent needle was replaced with a needle leading to a balloon, which was then filled and emptied 3x. After the final purge, the filled balloon was left to ensure the culture tube remained under positive pressure argon. The solution prepared in the scintillation vial was then taken up in a syringe which was then fitted with a 0.2 µm PTFE filter with a needle on the outlet and filtered into the culture tube. This culture tube was then transferred to the recirculating flow setup and clamped above a magnetic stir plate. Two units of FEP tubing were inserted into the vial – the inlet and outlet to the flow path. The inlet led to a Vici Valco positive displacement pump (previously described), which then lead to the large echem parallel plate reactor with 10 cm x 10 cm glassy carbon plates. The reaction was circulated through the system, and electricity was applied. The reactions were run at 4.5 V, constant voltage, switching polarity every 20 sec. After

12 hr of continuous run time, the reaction was stopped. The reactor setup was then flushed with 30 mL DMSO. The reactions were then poured into a 125 mL separatory funnel, to which 50 mL of water was added. This mixture was extracted 3 times with 20 mL dichloromethane, with the organic layers dried over magnesium sulfate. The combined organics were filtered, the residual drying agent rinsed with 30 mL dichloromethane, after which the solvent was removed by rotary evaporation. This crude mixture was deposited silica gel and dry loaded onto a column (20 cm diameter). The mixture was purified by silica gel chromatography using 20 % ethyl acetate in hexanes. This was not sufficient to fully purify the product, so all fraction containing the desired product were combined and concentrated, and chromatographed again using 1 % ethyl acetate in hexanes. The product was afforded as a white solid (14 mg, 25 %). The 1H and 13C data matched what has been previously reported.[13]

1H NMR (CDCl3, 500 MHz) δ 7.60 (d, J = 8.1 Hz, 4H), 7.27 (d, J = 7.8 Hz, 4H), 4.10 (s, 2H).

^{13}C NMR (126 MHz, $CDCl_3$) δ 144.80, 132.60, 129.71, 118.67, 110.82, 41.91.

Site Selectivity of Electrochemical Oxidation Case Study.

This dataset was downloaded using Reaxys, searching for “electrochemical oxidation” reactions. The original search was exported to give the raw dataset, after which all entries were manually curated to give the final dataset. During this process, only entries in which carbon atoms were oxidized were retained. Further, reactions involving hydrogen atom transfer catalysts were omitted. To identify the atom oxidized, the reactions were first atom mapped with RXNmapper.[14] After mapping, the reactions were manually examined to check for errors resulting from incomplete reaction SMILES. Then, the oxidation state change for each carbon atom was calculated by computing the oxidation state of each carbon in the products and reactants. Oxidation state was calculated according to the following guidelines: (1) oxidation state counting begins at zero, (2) if the carbon of interest is bound to a more electropositive atom, the oxidation state is decreased by one, (3) if the carbon of interest is bond to a more electronegative atom,

the oxidation state is increased by one, (4) multiple bonds to the same element are counted multiple times. For example, the carbon in formaldehyde has an oxidation state of 0; -1 from each bond to hydrogen and +1 for each bond to oxygen. By performing this calculation for each carbon in the product and reactant molecules, the ones for which the oxidation state increases from reactant to product are identified as oxidized centers. For the purpose of the classification problem, these atoms are assigned a value of “1” whereas the other atoms are assigned a value of “0”. For the atom numbered reactants, the assignment of which atoms are oxidized in the reaction (as well as the reaction category for the leave-one-group-out validation) can be found in the classifiedrxns.csv file. For mapped reaction smiles as well as the substructure surrounding the reactive atom, refer to the mapped_rxns_with_substructures.csv file.

For the DFT representation, structures were first prepared using RDKit by converting the smiles string of the reactant to a RDKit molecule object, then embedding 100 conformers per molecule using Chem.rdDistGeom.EmbedMultipleConfs (all other parameters left as defaults). The conformers were minimized with UFF and the lowest energy conformer for each molecule saved. For each molecule, the lowest energy conformer, as calculated with UFF in RDKit, was then minimized at the B3LYP/def2svp level of theory using Gaussian 16.[15] Following minimization, NBO calculations were performed on the singlet structure for the singlet, oxidized, and reduced species at the same level of theory. The NBO data was parsed using the NBO_Parser.py script provided in the supplementary data. Specifically, the features extracted are the energy and occupancy of the four highest orbitals for that atom (for example, if a bonding orbital is the highest energy orbital shared between two atoms, both atoms are assigned identical data for that orbital), the 1s orbital energy, the SOMO energy and occupancy, and the atomic number. For the Morgan fingerprints, atom level fingerprints were generated using RDKit with a radius of 2 and nBits=128. Notably, varying lengths for the fingerprints were tested (up to 1024 bits). These were evaluated on the basis of cross validated score using the four reaction groups used for training. In all cases, cross validated score was high and bit ranges from 128-1024 performed similarly; as such, the

representation with lowest dimensionality was used.

The modeling endeavors for the site selectivity prediction model was set up as a binary classification problem. Each atom was parameterized independently, containing only atomic properties. However, for the purpose of data partitioning, data partitioning was done per molecule (e.g., no molecules have atoms both in the train and in the test set). For each atom, the question is asked: is this atom oxidized in the course of the reaction or not? If it is, the atom is assigned a 1, if not it is assigned a 0. For model generation, both feedforward neural networks (implemented with PyTorch) and random forest models (implemented with SKlearn[16]) were tested. For the leave-one-group-out approach, models were evaluated using the 5-fold cross validated score of the four clusters used during model training. Random forest models gave higher scores with each descriptor set, with the final models using 1000 estimators and all other hyperparameters left as the default value in SKlearn (a threshold of 0.5 was used). For the final performance evaluation, the left-out groups were aggregated and precision and recall calculated. The confusion matrices of the left-out aggregate are also provided here. Also listed are True Positive Rates (TPR), True Negative Rates (TNR), and accuracy (F1). Additionally, the prediction probability was also returned – this number was used as an indication of how likely that center is to be oxidized.

A number of different features sets and modeling strategies were tested, which are briefly summarized here. Four different sets of descriptors were tested and compared via cross validation. DFT_1 refers to using only the electronic structure features mentioned above for the neutral structure. DFT_2 refers to the concatenated neutral and oxidized electronic structure features. DFT_3 refers to the concatenated neutral and reduced electronic features. DFT_4 refers to the concatenated neutral, reduced, and oxidized electronic structure features. Also tested was the use of a weighted loss function to account for the imbalance of training data between oxidized and not oxidized centers. A summary of the results using neural networks is as follows:

Table S3

Features	Precision**	Recall**	F1**	PR_AUC	Comments
DFT_1	0.300	0.319	0.309	0.245	Only Uses Neutral Electronic Structure
DFT_2	0.321	0.537	0.402	0.274	Uses Electronic Structure of Neutral Species and Radical Cation
DFT_3	0.322	0.449	0.375	0.274	Uses Electronic Structure of Neutral Species and Radical Anion
DFT_4	0.330	0.601	0.426	0.288	Uses Neutral Species, Radical Cation, and Radical Anion
DFT_4*	0.405	0.659	0.501	0.314	Includes Weighted Loss Function
Morgan + DFT_4*	0.257	0.619	0.363	0.228	Includes Weighted Loss Function
Morgan*	0.176	0.309	0.224	0.161	Includes Weighted Loss Function

** Threshold = 0.5

In addition to these efforts, random forest models were tested. In this case, the default random forest models using SKLearn and no class weighting outperformed the neural network models. The results of this model are as follows:

Table S4.

**DFT Vector**			
	Predicted 0	Predicted 1	Totals
Observed 0	4731	301	5032
Observed 1	146	307	453
			5485
**Precision**	**Recall**		
0.505	0.678		
**TNR**	**TPR**	**Accuracy**	
0.940	0.678	0.919	

Table S5

Reference 22 of Main Text			
	Predicted 0	Predicted 1	Totals
Observed 0	3631	1401	5032
Observed 1	251	202	453
			5485
**Precision**	**Recall**		
0.126	0.446		
**TNR**	**TPR**	**Accuracy**	
0.722	0.446	0.699	

Table S6

Morgan Fingerprints			
	Predicted 0	Predicted 1	Totals
Observed 0	4301	731	5032
Observed 1	328	125	453
			5485
**Precision**	**Recall**		
0.146	0.276		
**TNR**	**TPR**	**Accuracy**	
0.855	0.276	0.807	

Table S7

Morgan Fingerprint + DFT Vector			
	Predicted 0	Predicted 1	Totals
Observed 0	4186	846	5032
Observed 1	173	280	453
			5485
**Precision**	**Recall**		
0.249	0.618		
**TNR**	**TPR**	**Accuracy**	
0.832	0.618	0.814	

Unsupervised Embedding Process and Case Studies

Having developed an adequate atomic representation in the site selectivity prediction model, we next sought to convert the collection of atom-level properties into a fixed length, molecular vector. To achieve this task, we implemented graph2vec, using the atomic properties

as node attributes. Edges in the graph correspond to bonds, but no additional edge attributes were added. Because of the length and complexity of the atomic vector, decimal values were rounded to the tenths place and different, truncated atom vectors were tested. Additionally, hyperparameters for the embedding process were also optimized. The parameters optimized were the number of epochs, wl-iterations, learning rate, down sampling, and the dimensions of the embedded vector. For structure preparation, structures were first read into RDKit as a molecule object, followed by a rough conformer search beginning with embedding 100 conformers per molecule (the same process as used previously). The conformers were minimized with UFF and the lowest energy conformer for each molecule saved. These low energy conformers were then minimized at the B3LYP/def2svp level of theory using Gaussian 16. Following minimization, NBO calculations were performed on the base structure, oxidized structure, and reduced structure at the same level of theory.

Some justification of the level of theory is necessary. A very reasonable concern is that the B3LYP/def2svp level of theory is not sufficient for calculating accurate electronic structure properties. However, although error is present, we reasoned this error would be systematic. As such, it would be strongly correlated with values calculated at higher levels of theory. If this is true, the predictive performance of the resulting models would be expected to be the same. To test this hypothesis, we selected 5 molecules (4-chloro aniline, 2-methyl butadiene, DMAP, 2-methylindole, and 2-methylfuran) and recalculated the NBO features used in this study at the B2PLYP/def2TZVPP level of theory. The results are depicted in Figure S2. The R^2 between the features calculated with the two level of theories is 0.96 – given the strong correlation, we felt it reasonable to use the lower level of theory. Notably, we do not argue that this observation is generally true, simply that it is true in this specific case.

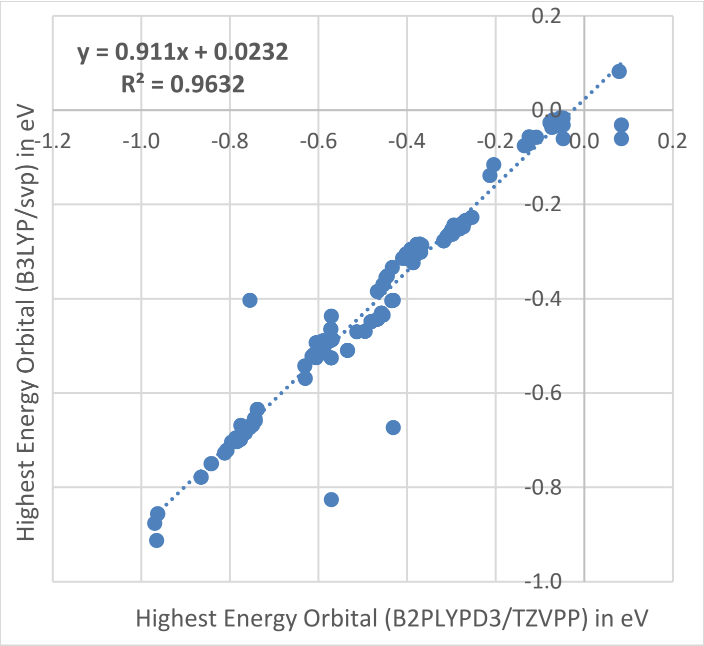

Figure S2. Energy of highest energy atomic orbital for neutral, oxidized, and reduced forms of representative models calculated at the B3LYP/SVP level of theory and the B2PLYPD3/TZVPP level of theory.

The process of converting the atom-level properties to molecule-level representations is relatively straightforward using graph2vec, and the documentation provided on the corresponding github page (https://github.com/benedekrozemberczki/graph2vec) makes it very easy to implement this approach. The main challenge in implementation is to convert the atomic properties into a format that is a valid input into the package. To do this, we have written a number of scripts, which are provided with this publication. First, the relevant NBO data must be collected from the DFT calculations – for this purpose, we wrote a script for parsing the relevant NBO information. The output of this is a .csv file of in which the first column contains the molecule label and atom number for a particular atom in the format moleculelabel_atom#, and subsequent entries on that line are atom properties. For the DFT properties, bond properties are localized to atoms. This means that if the highest energy occupied orbital for a pair of atoms is shared for a pair of atoms (for example, the pi bonding orbital is the highest energy orbital that can be localized to those atoms), both atoms receive properties from that bonding orbital (e.g., the occupancy and energy values for those two atoms will be the same for that feature).

It is worth noting that the DFT properties used as input are rounded to the tenths place before being used as input to graph2vec. The rational behind this is that the first step of the graph2vec process involves building a "vocabulary", which is the sampling and relabeling of all subgraphs. This step relates to the embedding process because it defines the prediction task from which the embedding is extracted. After sampling and relabeling subgraphs, a skip gram model is trained which (simply put) predicts which subgraphs are present in the graph. The embedding is then extracted from the hidden layer of this model. By rounding the DFT data, we are essentially making the subgraphs more general. If all subgraphs are too specific, molecules share no or very few common subgraphs – consequently, the embedding loses the ability to distinguish similar or dissimilar molecules because they all contain different subgraphs. Rounding makes the subgraphs more general and was necessary to improve performance.

Note that the molecule labels must be integer values for input into graph2vec – in some cases, we had to change the atom labels to accommodate this. Next, the molecular graphs must be constructed. These are stored as .json files. The .csv file containing the NBO information generated from the NBO parser is cross referenced either with the SMILES or a .mol2 file read in with RDKit (note: consistent atom numbering is imperative). Using this information, the connectivity can be established. Because there are no edge properties used in the representation, only connectivity is recorded. The node properties are then the atomic properties. This information is common to all case studies which use the graph2vec framework.

For the ionization potential, nucleophilicity, and enantioselectivity case studies, all data not included in the test set was used in 3-fold cross validation with each feature set. The hyperparameter and atom level feature sets with the best cross validated score were then used as the final descriptor sets, which were used to evaluate the test set. For the reactivity prediction study using the data generated on the automated platform, the hyperparameter combination and atom-level features which were best for ionization potential were also used without further optimization. It is probable that the accuracy of this model could improve with further optimization,

but we instead chose to show the generality of the method by making superior models without extensive hyperparameter optimization. The approach for hyperparameter optimization was a random search – this search was executed by making a batch file which, when executed, generates descriptor files for each molecule in the dataset. A script for generating the fingerprint search space is given in randomly_search_print_space.py in the supplemental files. This script references molecular graphs stored in .json files. A script for constructing .json files from .mol2 files is provided in the supplementary files called json_from_mols.py, along with the requisite DFT data (stored in BINOL_1.csv) and .mol2 files. For every case study, the best atom level features were the highest energy orbital assigned to that atom for the neutral species, the radical anion, and the radical cation. For ionization potential and reactivity prediction case studies, the hyperparameters used were 14 epochs, 128 dimensions, 7 weisfeler lehman iterations, minimum count of 4, learning rate of 0.075, and down sampling set to 0.00075. For the nucleophilicity case study, the hyperparameters used were 305 epochs, 64 dimensions, 3 weisfeler lehman iterations, minimum count of 1, learning rate of 0.0001, and down sampling set to 0.1. For the enantioselectivity dataset, catalyst structures were represented with the DFT embedded vector using the following hyperparameters: 430 epochs, 256 dimensions, 166 weisfeler lehman iterations, minimum count of 1, learning rate of 0.025, and downsampling set to 0.075. The substrate descriptors were identical to those used in a prior study to facilitate a direct comparison of catalyst representations.[17] Notably, the dense vector output is scaled such that the sum of the vector is equal to 1. For model development, both the ionization potential and the nucleophilicity prediction models used Projection to Latent Structure (PLS) models with three latent variables. The logic for this choice was that we simply wanted to test if the model performed as good or better than other methods. Further, given that the models are inherently extrapolating, we reasoned a simple model might perform better than more complex nonlinear models. It is possible that finetuning of the model choice could give some improvements to accuracy; however, the accuracy was sufficient to conclude that the current representation is valid and generalizes well

enough to proceed to the main purpose of the work, i.e. reactivity prediction. For the enantioselectivity case study, gradient boosting regressors with a random hyperparameter search were selected. This choice was made to facilitate direct comparisons to prior work.[17] The search was performed using the randomsearchcv function in rdkit, and the parameter space is as follows: 'learning_rate' was sampled from a uniform distribution ranging 0 to 1, 'n_estimators' was sampled from a range of integers from 1 to 999, 'subsample' was sampled from a uniform distribution ranging 0 to 1, 'min_samples_split' was set to 0.1, 0.2, 0.3, 0.4, or 0.5, 'max_depth' was set to an integer value ranging from 1 to 50, and 'max_features' was sampled from a uniform distribution ranging 0 to 1.

The reactivity prediction model used a ridge classification model with model parameters kept as their default values. All modeling was implemented using SKLearn. The DFT-embedded fingerprint was used directly as input into the models. Ten different models were constructed with different, random train / test partitioning. Each of the ten models give a binary output for each molecule; -1 if the molecule is predicted to be unreactive, 1 if the molecule is predicted to be reactive. The output is then the average of all predictions, where any positive outcome is predicted to be reactive. Because the output is an average of an ensemble, values ranging from 1 to -1 are possible. A value of 1 means that every model "voted" that that molecule should be reactive – this is viewed as a high confidence prediction. A value of 0 means five models "voted" the molecule to be reactive and five "voted" that molecule to be unreactive. In this case, the prediction is uncertain. Consequently, as values become more positive, confidence increases that the molecule will be reactive and as values become more negative confidence increases that the molecule will be unreactive. The entire dataset of ~38k molecules was fed through this ensemble, and the predictions are available in the prediction_summary.xlsx file. We hope other researchers find this document useful for designing new electrochemical reactions.

Finally, many other modeling types of varying levels of complexity were tested for the reactivity prediction model. In all cases, 116 data points were used for model training and 3-fold

cross validation, with accuracy as the scoring metric. The remaining 25 were held out as an external test set. The ridge classifier exhibited the best performance, as was thus used in the discovery workflow. The summary of this data is present in table S8.

Table S8. Comparison of Model Performance (results are accuracy reported as decimal value). SVC is Support Vector Classifier, KNN is K-Nearest Neighbor classifier, RFC is Random Forest Classifier, GPC is Gaussian Process Classifier, MLPClassifier is Multi-Layer Perceptron Classifier, QDA is Quadratic Descriminant Ananlysis, and GBC is Gradient Boosting Classifier.

**Model**	Method	1	2	3	4	5	6	7	8	9	10	**Mean**
**RidgeClassifierCV**	Cross Val Accuracy	0.73	0.71	0.69	0.72	0.74	0.71	0.74	0.73	0.75	0.71	**0.73**
	Test Accuracy	*0.76*	*0.76*	*0.80*	*0.80*	*0.64*	*0.76*	*0.68*	*0.80*	*0.80*	*0.64*	***0.74***
**SVC_linear**	Cross Val Accuracy	0.63	0.71	0.61	0.61	0.69	0.70	0.69	0.69	0.66	0.67	**0.67**
	Test Accuracy	*0.73*	*0.62*	*0.81*	*0.73*	*0.77*	*0.69*	*0.69*	*0.69*	*0.73*	*0.62*	***0.71***
**SVC_rbf**	Cross Val Accuracy	0.68	0.66	0.65	0.68	0.71	0.62	0.73	0.67	0.68	0.68	**0.68**
	Test Accuracy	*0.73*	*0.77*	*0.77*	*0.73*	*0.69*	*0.77*	*0.77*	*0.62*	*0.69*	*0.54*	***0.71***
**SVR_poly**	Cross Val Accuracy	0.60	0.61	0.62	0.63	0.63	0.58	0.59	0.62	0.60	0.65	**0.61**
	Test Accuracy	*0.73*	*0.58*	*0.65*	*0.69*	*0.62*	*0.77*	*0.73*	*0.50*	*0.65*	*0.54*	***0.65***
**KNN_dist**	Cross Val Accuracy	0.72	0.70	0.70	0.76	0.68	0.69	0.70	0.74	0.68	0.74	**0.71**
	Test Accuracy	*0.62*	*0.69*	*0.73*	*0.62*	*0.65*	*0.69*	*0.85*	*0.73*	*0.69*	*0.62*	***0.69***
**KNN_uniform**	Cross Val Accuracy	0.72	0.70	0.70	0.76	0.68	0.69	0.70	0.74	0.68	0.74	**0.71**
	Test Accuracy	*0.62*	*0.69*	*0.77*	*0.62*	*0.65*	*0.69*	*0.85*	*0.77*	*0.73*	*0.65*	***0.70***
**RFC**	Cross Val Accuracy	0.64	0.73	0.72	0.75	0.70	0.70	0.75	0.73	0.70	0.72	**0.71**
	Test Accuracy	*0.69*	*0.69*	*0.77*	*0.69*	*0.62*	*0.69*	*0.77*	*0.46*	*0.73*	*0.62*	***0.67***
**GaussianNB**	Cross Val Accuracy	0.64	0.61	0.75	0.58	0.65	0.57	0.67	0.65	0.59	0.64	**0.63**
	Test Accuracy	*0.77*	*0.69*	*0.69*	*0.58*	*0.46*	*0.58*	*0.77*	*0.38*	*0.62*	*0.46*	***0.60***
**GPC**	Cross Val Accuracy	0.61	0.70	0.68	0.74	0.74	0.67	0.69	0.81	0.69	0.69	**0.70**
	Test Accuracy	*0.85*	*0.65*	*0.77*	*0.65*	*0.62*	*0.81*	*0.85*	*0.65*	*0.77*	*0.69*	***0.73***
**AdaBoostClassifier**	Cross Val Accuracy	0.64	0.59	0.63	0.62	0.74	0.64	0.66	0.73	0.67	0.65	**0.66**
	Test Accuracy	*0.69*	*0.65*	*0.73*	*0.65*	*0.50*	*0.58*	*0.77*	*0.54*	*0.85*	*0.69*	***0.67***
**MLPClassifier**	Cross Val Accuracy	0.74	0.75	0.70	0.72	0.74	0.70	0.71	0.69	0.75	0.69	**0.72**
	Test Accuracy	*0.73*	*0.65*	*0.77*	*0.81*	*0.69*	*0.65*	*0.85*	*0.54*	*0.69*	*0.73*	***0.71***
**QDA**	Cross Val Accuracy	0.51	0.55	0.61	0.52	0.54	0.54	0.52	0.51	0.55	0.55	**0.54**
	Test Accuracy	*0.62*	*0.46*	*0.62*	*0.54*	*0.54*	*0.58*	*0.69*	*0.50*	*0.58*	*0.69*	***0.58***
**GBC**	Cross Val Accuracy	0.63	0.63	0.70	0.61	0.66	0.70	0.69	0.69	0.72	0.69	**0.67**
	Test Accuracy	*0.69*	*0.54*	*0.69*	*0.85*	*0.65*	*0.73*	*0.81*	*0.62*	*0.77*	*0.73*	***0.71***

Large Scale DFT Dataset Calculation and Reactivity Prediction

In order to evaluate new potential reactants, a large dataset of molecules for which NBO properties were calculated was required. For this task, we first downloaded the QM7b dataset, then added 13,344 molecules randomly selected from the QM9 dataset, and then randomly selected an additional 17,894 molecules from the GDB-17 dataset.[18-20] Finally, a small number (873) molecules were added which contain elements not found in these datasets, including Si, P, S, Li, Na, K, Br, and I. These molecules were generated using ccheminfolib.[21] For molecules from the QM9 and QM7b dataset, the existing geometries were used as starting geometries. For the molecules generated using ccheminfolib, conformer searches were performed using Macromodel with the OPLS3e force field and no solvation.[22] For molecules selected from the GDB-17 dataset, the smiles were converted to molecule objects in RDKit. For each molecule, 100 conformers were embedded and minimized with MMFF. Then, the lowest energy conformer was selected as a starting structure (this is the same workflow as previously described). All of these structures were then minimized at the B3LYP/def2svp level of theory. Structures that failed to converge or contained imaginary frequencies after this step were eliminated. All other structures were then subjected to NBO calculations at the same level of theory for the singlet structure (same number of electrons as the optimization job), the reduced analog, and the oxidized analog. Molecules for which these three jobs completed successfully were retain and others were discarded. The NBO data obtained from these jobs were combined with those from the other case study and passed through the embedding process following the guidelines listed in the previous section to yield a total of 38,865 embeddings. Beyond the scope of this study, we reasoned that this large dataset of electronic structure data could be of interest to the broader community. These files will be made available upon request. Finally, predictions were made for the entire library. Namely, the results of the ten random partitions were averaged to produce a number ranging from 1 to -1 (reactive and unreactive, respectively). In this case, more positive numbers are higher confidence

predictions and more negative numbers are lower confidence predictions. The predictions are located in the prediction_summary.xlsx file, and the keys and labels (connecting prediction and structure file to smiles) are in the library_keys.xlsx and fullsmi.xlsx files, respectively. All of these files are in the Reactivity Prediction/ folder of the supplementary files.

Detailing reactions were selected from this set to be tested experimentally, we attempted to approach this in a way similar to how an experimentalist might use the files we provide for new reaction design. First, the reactions were sorted by the predicted value (the value ranging from 1 to -1, in which 1 is voted as reactive by all models and -1 is voted as unreactive by all models). Of the 38865 molecules for which predictions were made, only 825 are predicted to be reactive. This is the equivalent of preemptively omitting 38040 possibilities in >5 minutes of wait time, returning a set of molecules that is much more manageable to expert evaluation. Of these, a qualitatively diverse set was identified manually, for which commercially available anaologs were selected and evaluated. Notably, work is ongoing to automate this process by coupling this workflow to a database of commercially available molecules.

When comparing the 141 “in-stock” molecules to the total chemical space from the ~38k member dataset, the “in-stock” molecules give surprisingly good coverage considering they were not systematically selected (Figure S3). However, clearly the maximum density of the “in-stock” molecules is shifted from the *in silico* molecules. Further, some points appear to be extrapolations - we anticipate our ensembling approach gives some reliable metric of confidence and would flag these predictions as unreliable, but we are interested further studying in impact that initial sampling has on model reliability and discovery rate.

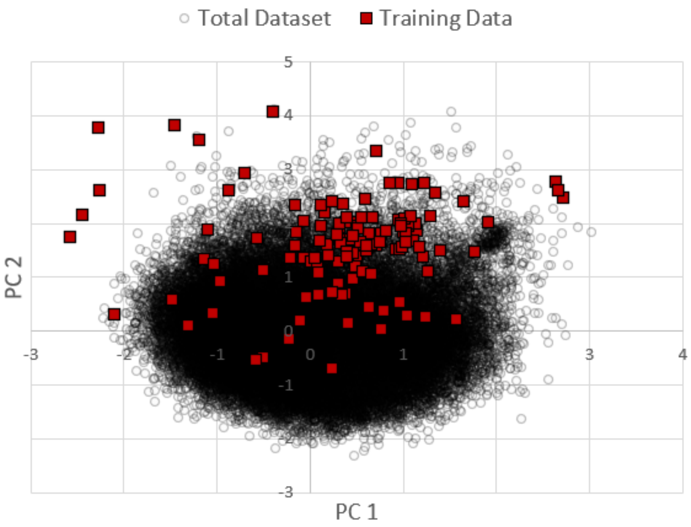

Figure S3. Overlayed Chemica Space of training set and 38k *in silico* dataset.

NMR Data

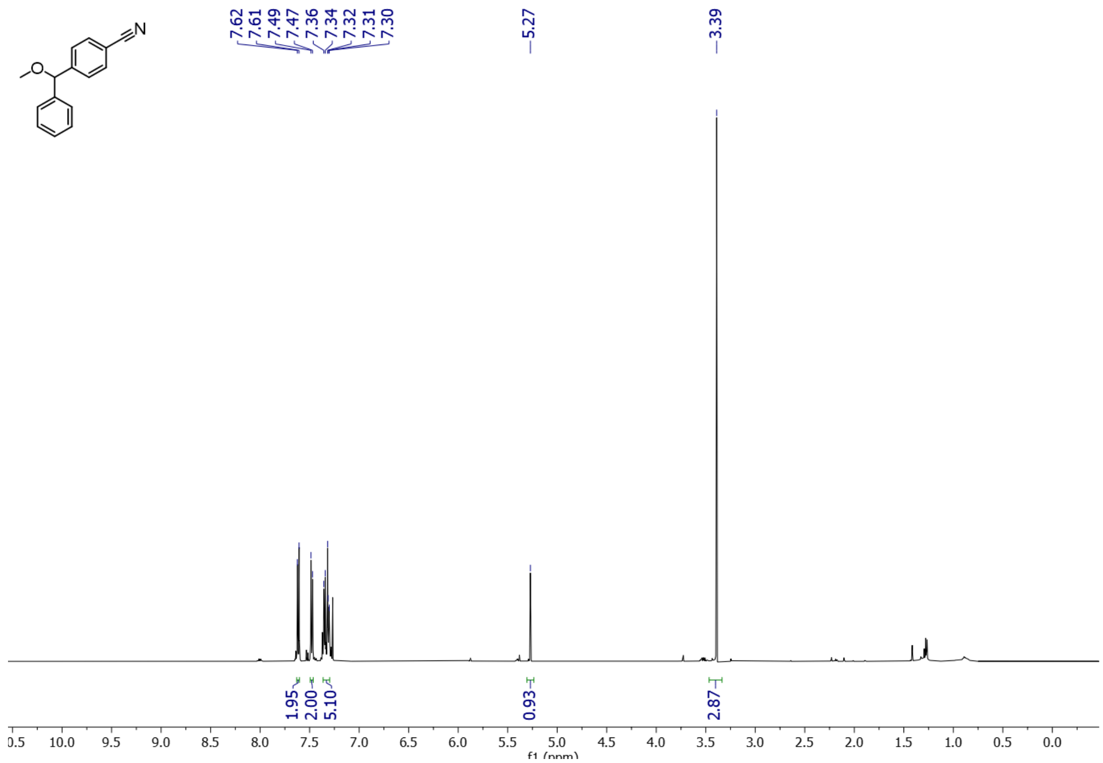
N
O
7.62
7.61
7.49
7.47
7.36
7.34
7.32
7.31
7.30
5.27
3.39
1.95
2.00
5.10
0.93
2.87
10.0 9.5 9.0 8.5 8.0 7.5 7.0 6.5 6.0 5.5 5.0 4.5 4.0 3.5 3.0 2.5 2.0 1.5 1.0 0.5 0.0
f1 (ppm)

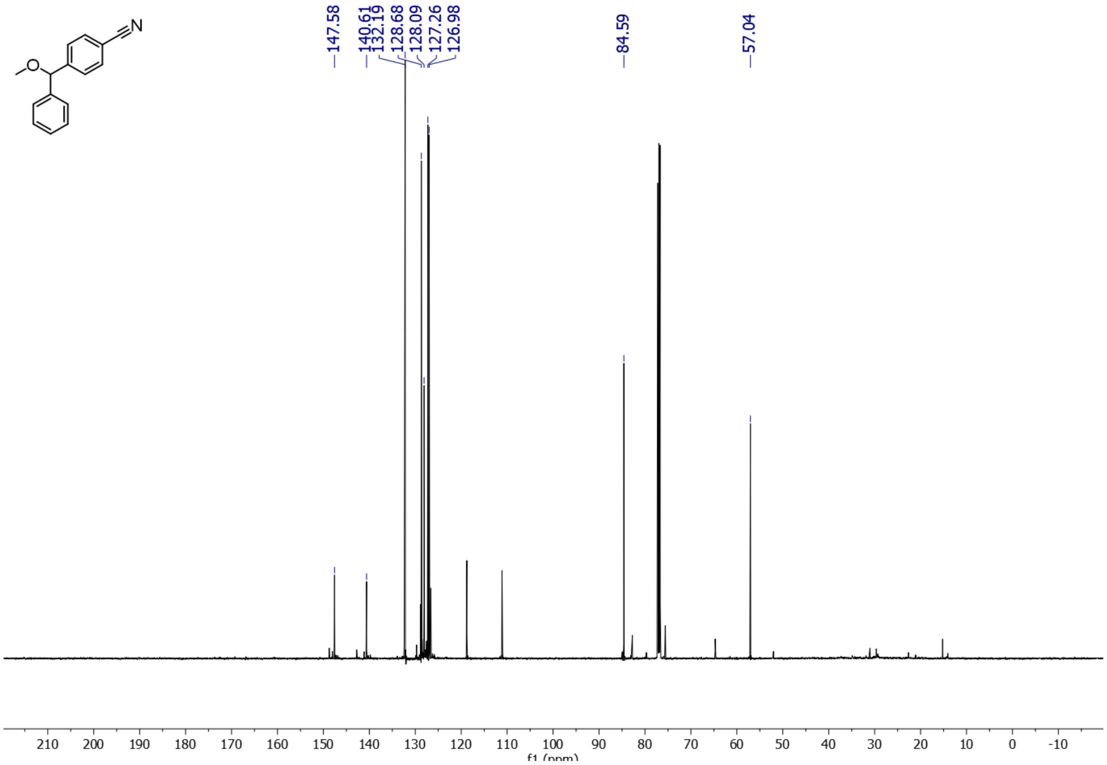
N
O
147.58
140.61
132.19
128.68
128.09
127.26
126.98
84.59
57.04
210 200 190 180 170 160 150 140 130 120 110 100 90 80 70 60 50 40 30 20 10 0 -10
f1 (ppm)

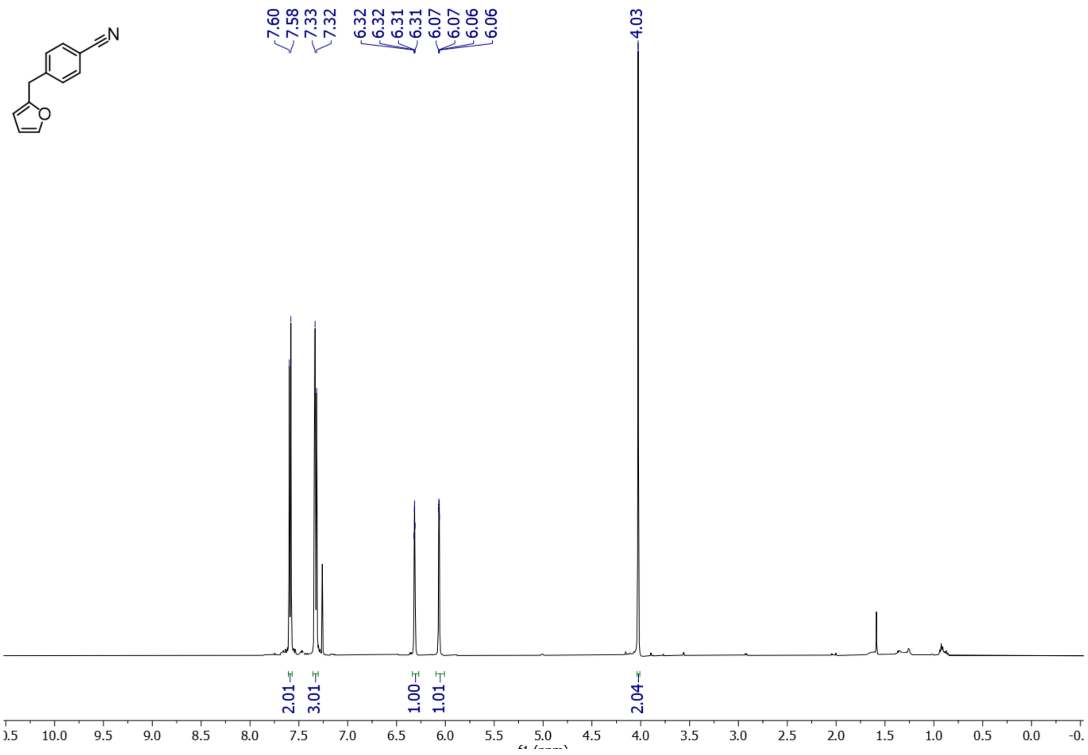
N
O
7.60
7.58
7.33
7.32
6.32
6.32
6.31
6.31
6.07
6.07
6.06
6.06
4.03
2.01
3.01
1.00
1.01
2.04

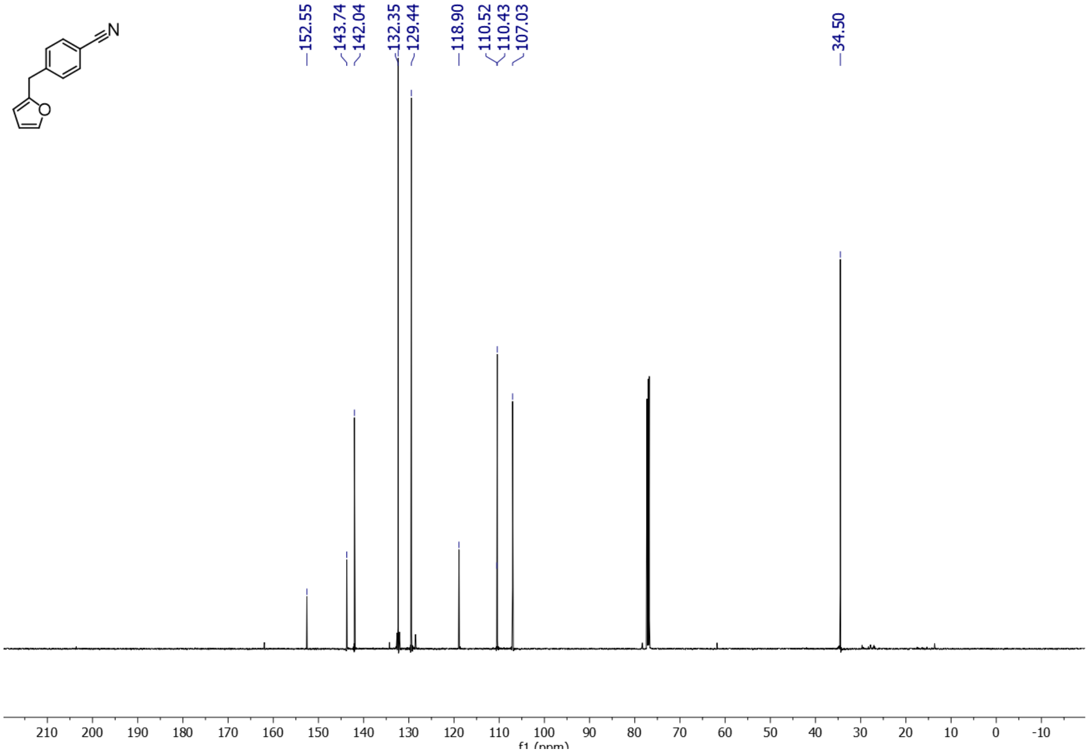
N
O
152.55
143.74
142.04
132.35
129.44
118.90
110.52
110.43
107.03
34.50
f1 (ppm)

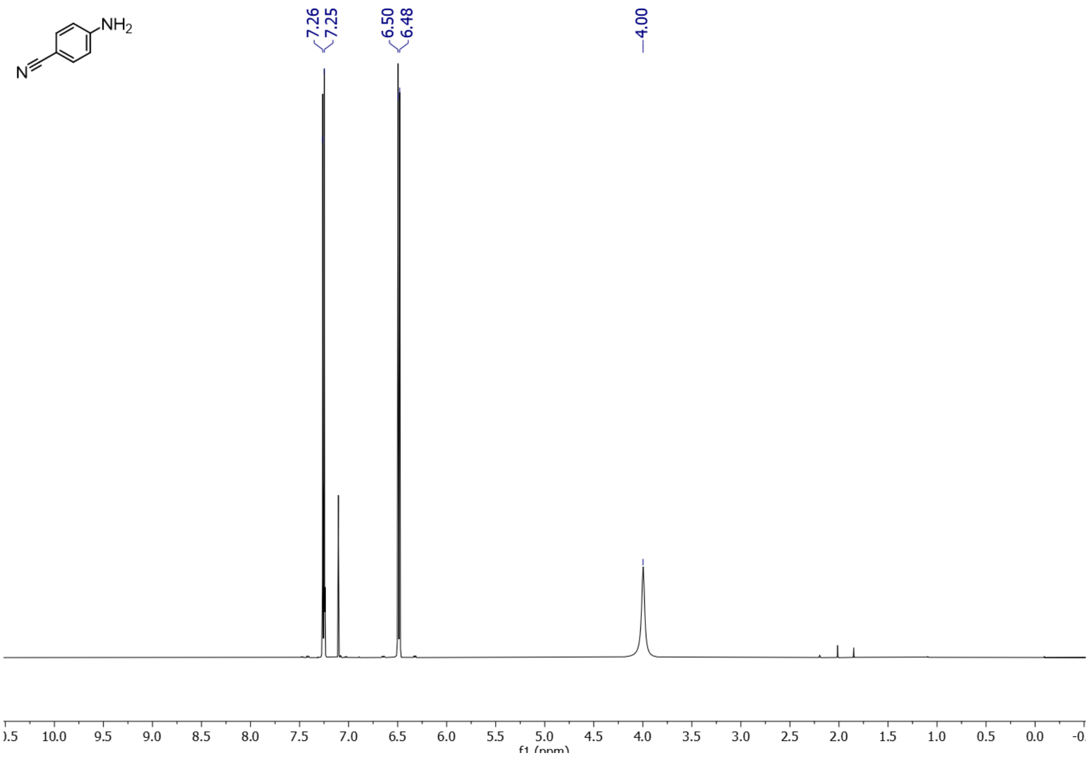
NH2
N
7.26
7.25
6.50
6.48
4.00
10.0 9.5 9.0 8.5 8.0 7.5 7.0 6.5 6.0 5.5 5.0 4.5 4.0 3.5 3.0 2.5 2.0 1.5 1.0 0.5 0.0

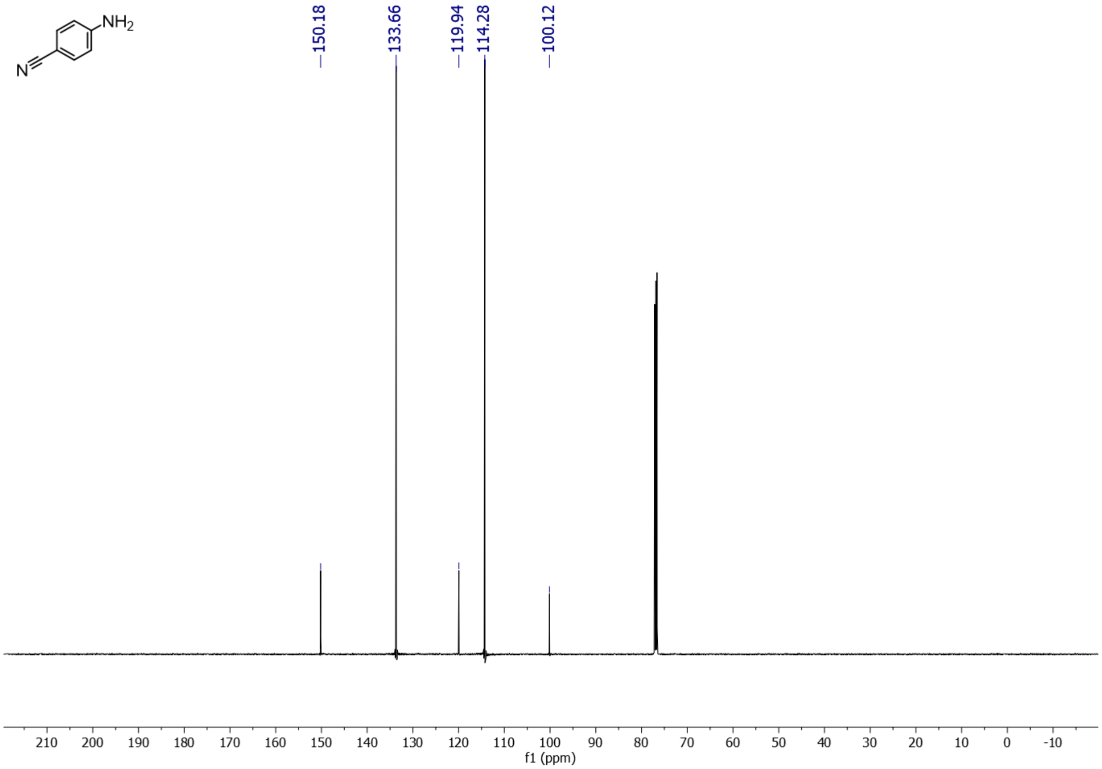
NH2
N
150.18
133.66
119.94
114.28
100.12
210 200 190 180 170 160 150 140 130 120 110 100 90 80 70 60 50 40 30 20 10 0 -10
f1 (ppm)

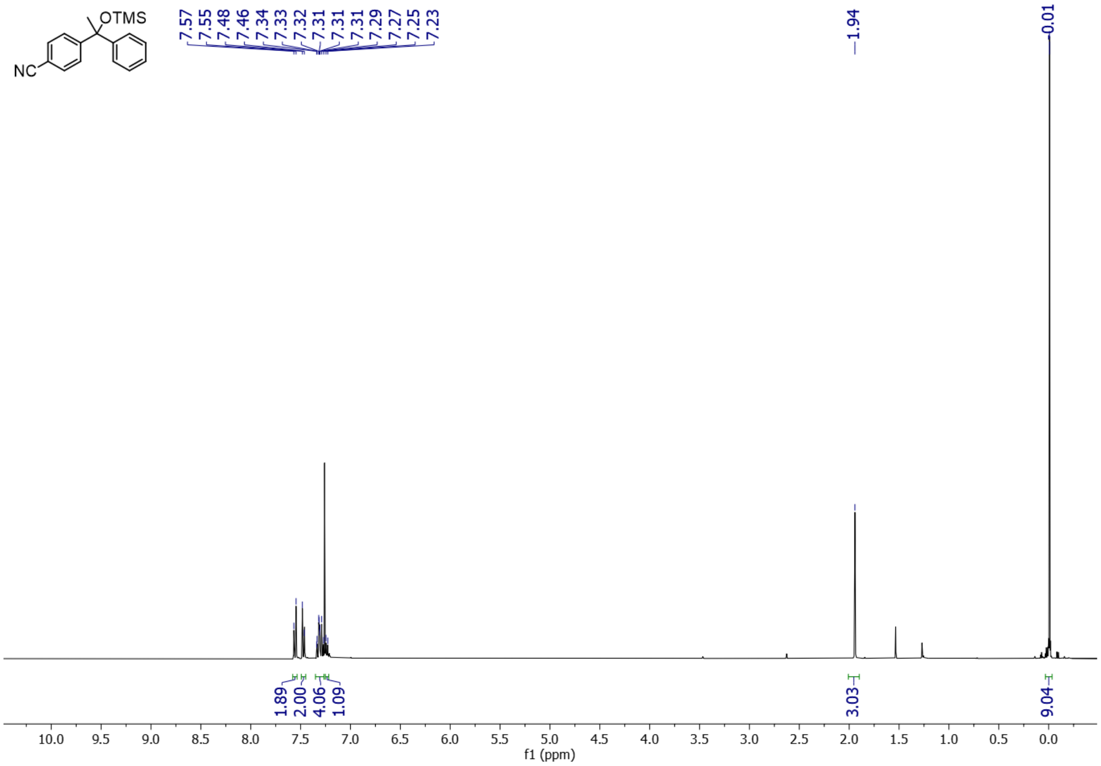
OTMS
NC
7.57
7.55
7.48
7.46
7.34
7.33
7.32
7.31
7.31
7.31
7.29
7.27
7.25
7.23
1.94
0.01
1.89
2.00
4.06
1.09
3.03
9.04
f1 (ppm)

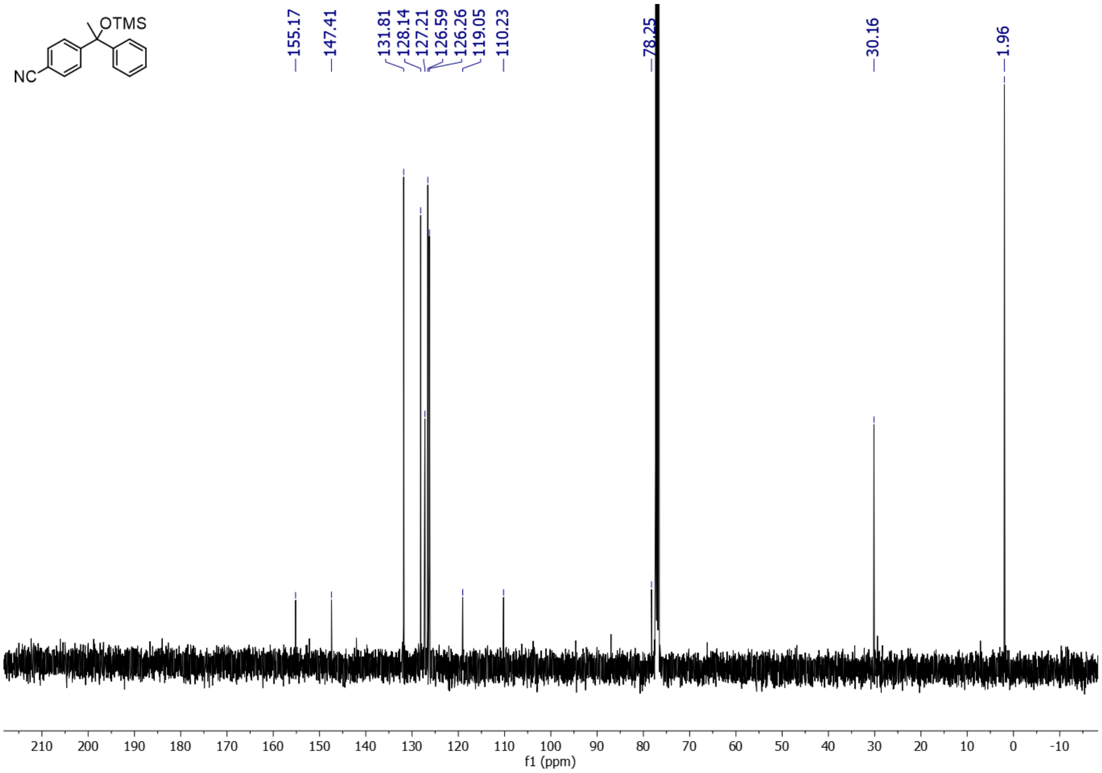
OTMS
NC
155.17
147.41
131.81
128.14
127.21
126.59
126.26
119.05
110.23
78.25
30.16
1.96
f1 (ppm)

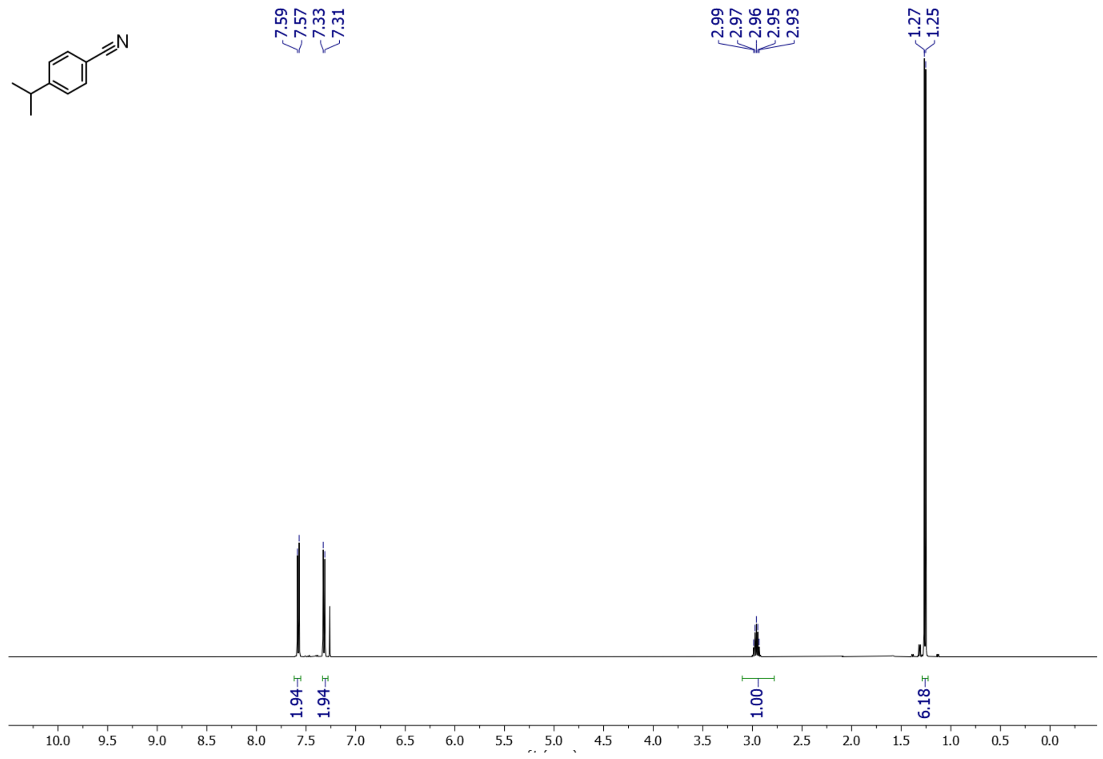
N
7.59
7.57
7.33
7.31
2.99
2.97
2.96
2.95
2.93
1.27
1.25
1.94
1.94
1.00
6.18
10.0 9.5 9.0 8.5 8.0 7.5 7.0 6.5 6.0 5.5 5.0 4.5 4.0 3.5 3.0 2.5 2.0 1.5 1.0 0.5 0.0

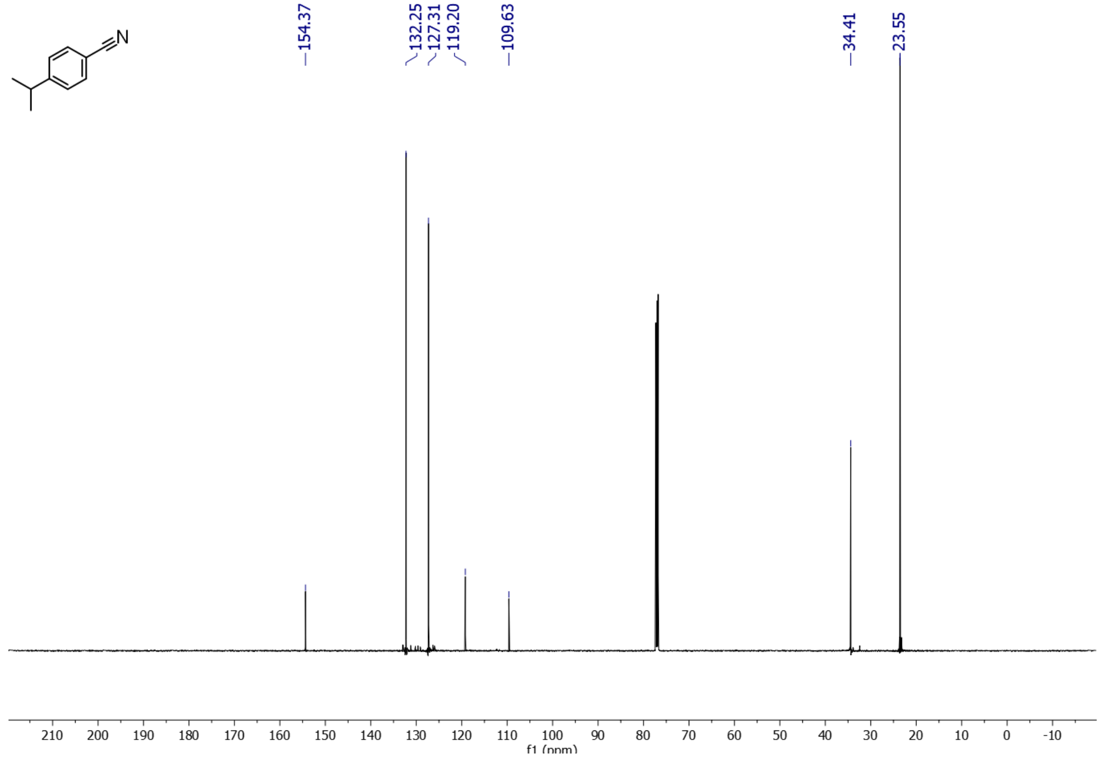
N
154.37
132.25
127.31
119.20
109.63
34.41
23.55
210 200 190 180 170 160 150 140 130 120 110 100 90 80 70 60 50 40 30 20 10 0 -10
f1 (ppm)

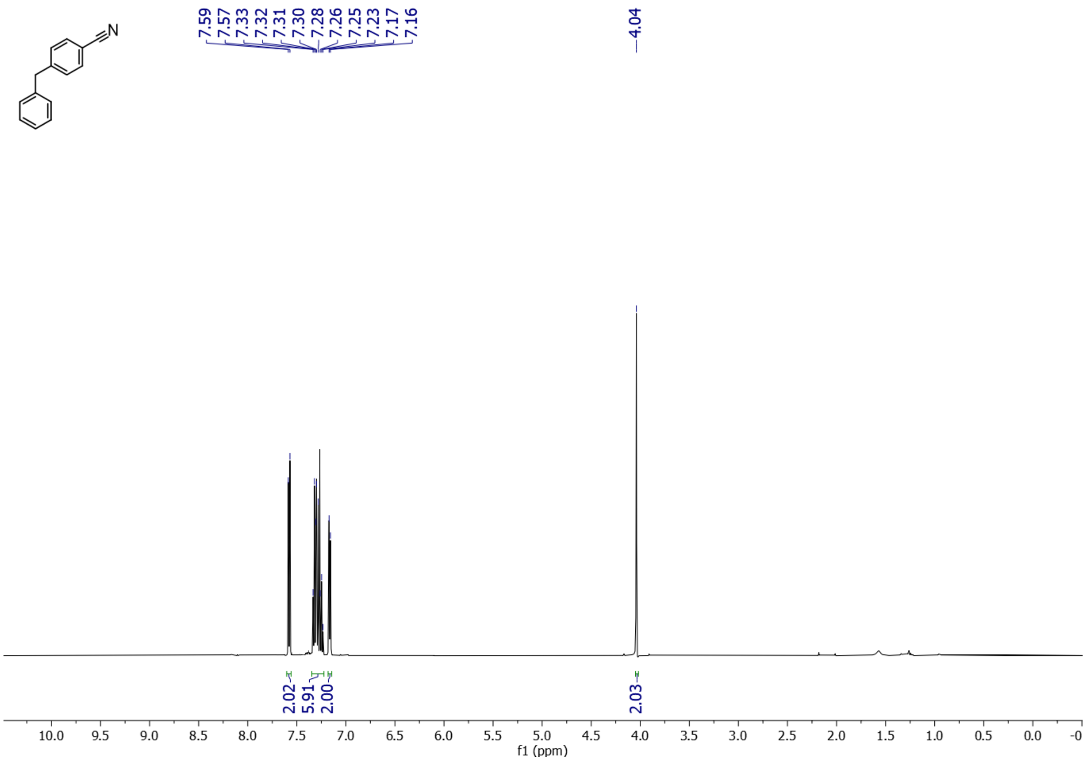
N
7.59
7.57
7.33
7.32
7.31
7.30
7.28
7.26
7.25
7.23
7.17
7.16
4.04
2.02
5.91
2.00
2.03
10.0 9.5 9.0 8.5 8.0 7.5 7.0 6.5 6.0 5.5 5.0 4.5 4.0 3.5 3.0 2.5 2.0 1.5 1.0 0.5 0.0 -0
f1 (ppm)

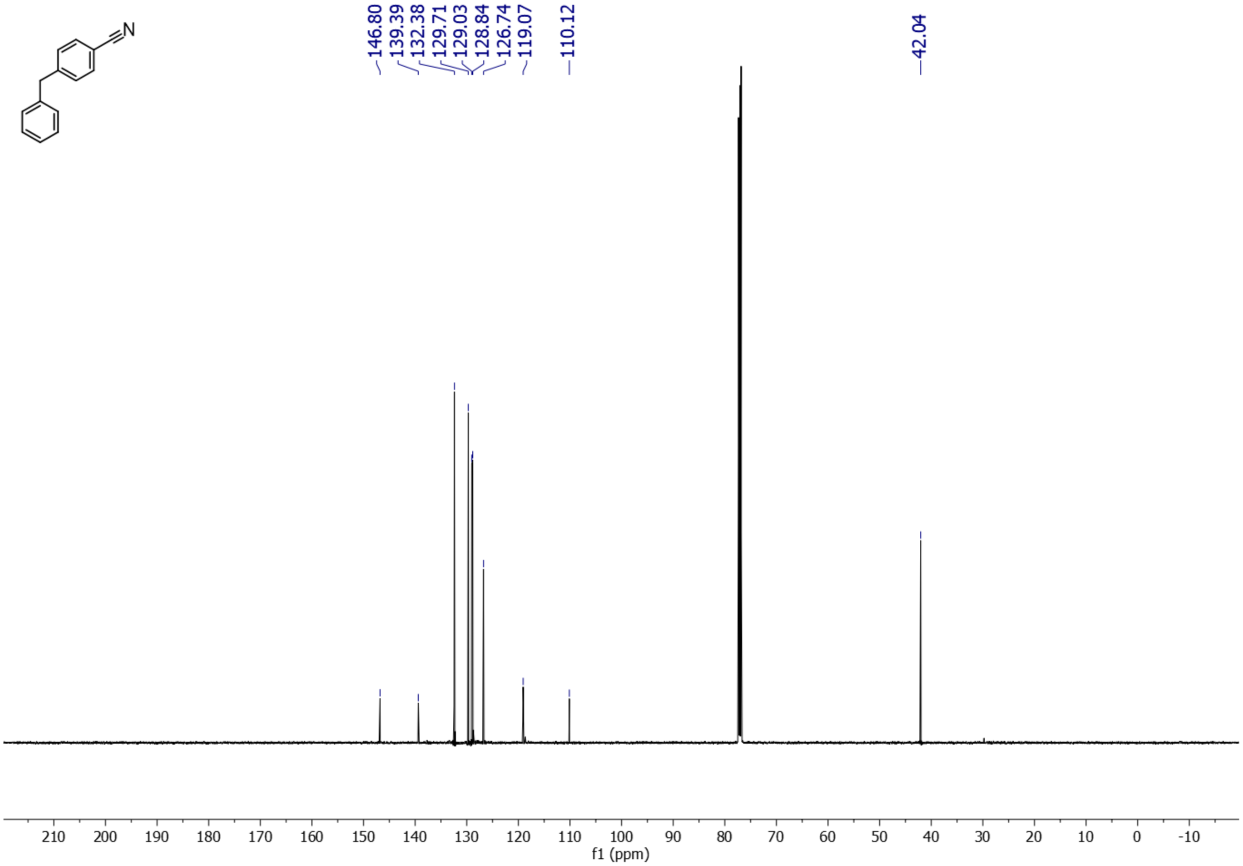
N
146.80
139.39
132.38
129.71
129.03
128.84
126.74
119.07
110.12
42.04
210 200 190 180 170 160 150 140 130 120 110 100 90 80 70 60 50 40 30 20 10 0 -10
f1 (ppm)

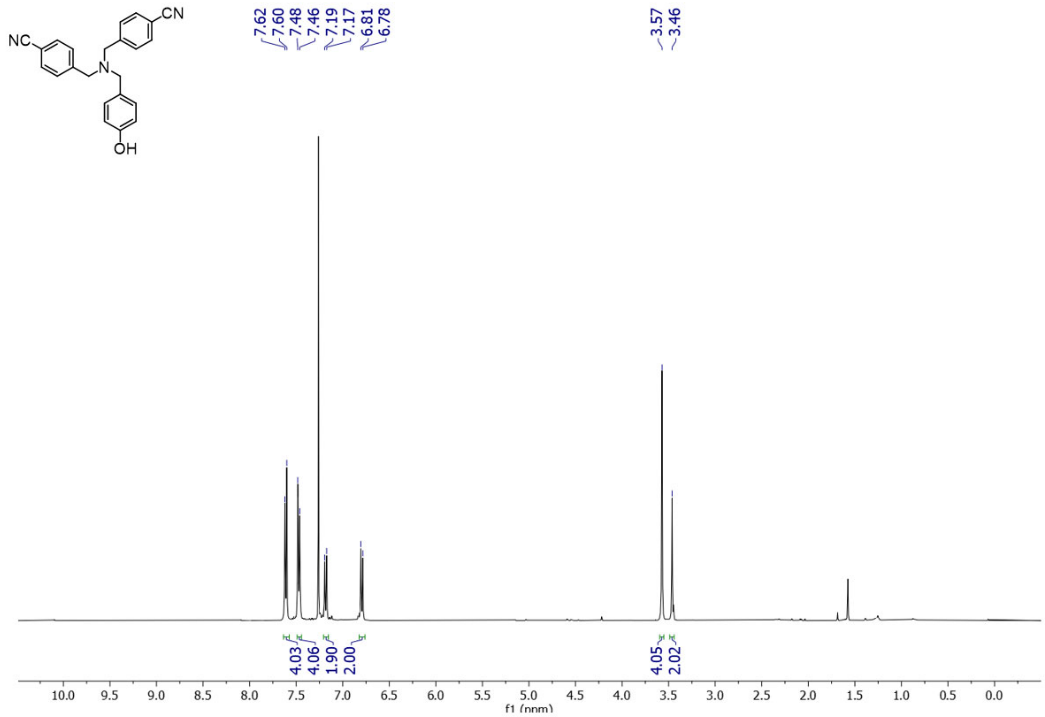
NC
CN
N
OH
7.62
7.60
7.48
7.46
7.19
7.17
6.81
6.78
3.57
3.46
4.03
4.06
1.90
2.00
4.05
2.02
10.0 9.5 9.0 8.5 8.0 7.5 7.0 6.5 6.0 5.5 5.0 4.5 4.0 3.5 3.0 2.5 2.0 1.5 1.0 0.5 0.0
f1 (ppm)

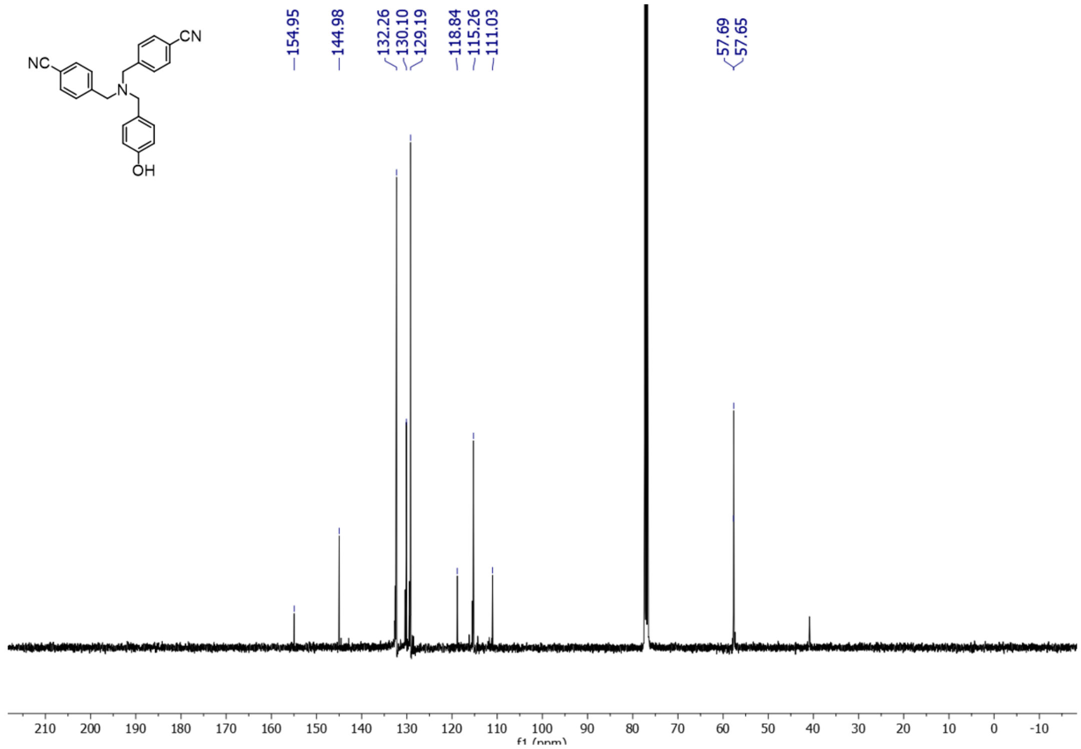
NC
CN
N
OH
154.95
144.98
132.26
130.10
129.19
118.84
115.26
111.03
57.69
57.65
210 200 190 180 170 160 150 140 130 120 110 100 90 80 70 60 50 40 30 20 10 0 -10
f1 (ppm)

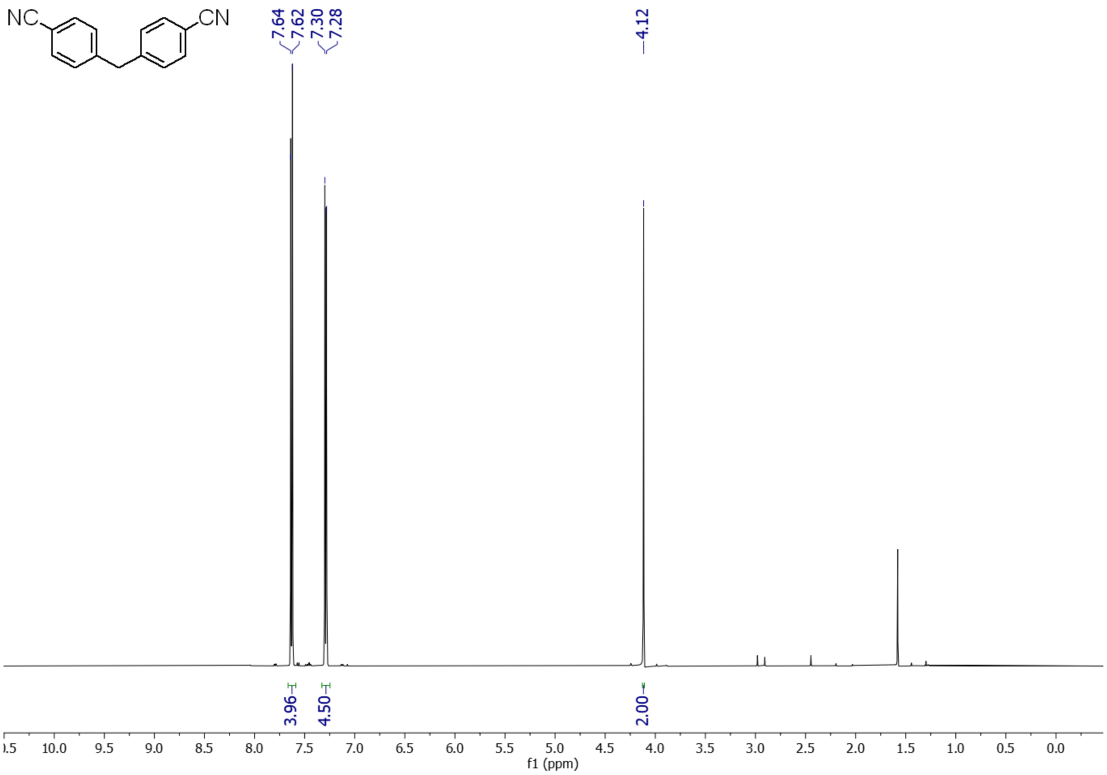

NC
CN
7.64
7.62
7.30
7.28
4.12
3.96
4.50
2.00
10.0 9.5 9.0 8.5 8.0 7.5 7.0 6.5 6.0 5.5 5.0 4.5 4.0 3.5 3.0 2.5 2.0 1.5 1.0 0.5 0.0
f1 (ppm)

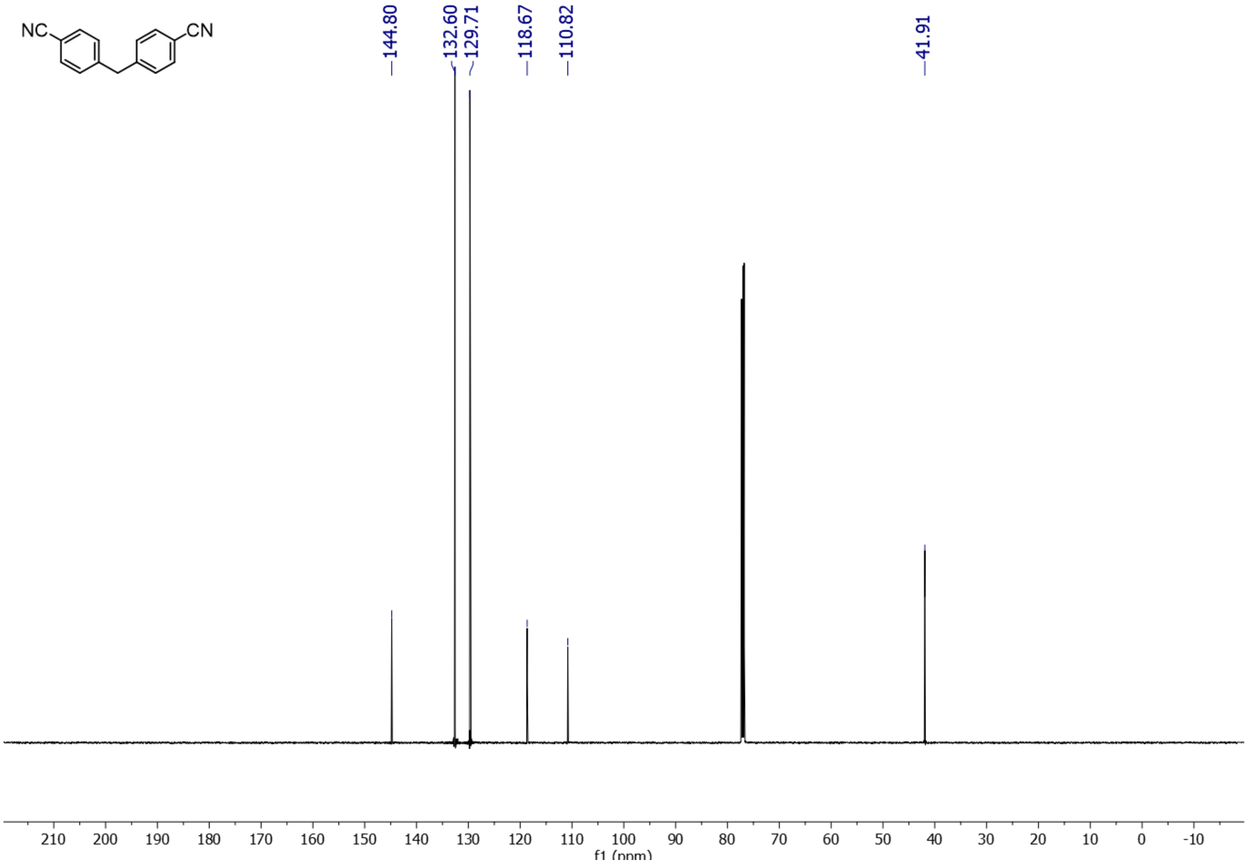

NC
CN
144.80
132.60
129.71
118.67
110.82
41.91
210 200 190 180 170 160 150 140 130 120 110 100 90 80 70 60 50 40 30 20 10 0 -10

References

[1] Mo, Y.; Rughoobur, G.; Nambiar, A.M.K.; Zhang, K.; Jensen, K. A Multifunctional Microfluidic Platform for High-Throughput Experimentation of Electroorganic Chemistry. *Angew. Chem. Int. Ed.* **2020**, *59*, 20890-20894.

[2] Mo, Y.; Lu, Z.; Rughoobur, G.; Patil, P.; Gershenfeld, N.; Akinwande, A.I.; Buchwald, S.L.; Jensen, K.F. Microfluidic Electrochemistry for Single-Electron Transfer Redox-Neutral Reactions. *Science*. **2020,** *368*, 1352-1357.

[3] Nandiwale *et al.* *React. Chem. Eng***.** **2022**, *7*, 1315-1327

[4] Matsumoto, K.; Toubaru, Y.; Tachikawa, S.; Miki, A.; Sakai, K.; Korki, S.; Hirokane, T.; Shindo, M.; Yoshida, M. Catalytic and Aerobic Oxidative Biaryl Coupling of Anilines Using a Recyclable Heterogeneous Catalyst for Synthesis of Benzidines and Bicarbazoles.

[5] Yuan, Y.; Shi, X.; Liu, W. Transition-Metal_Free, Chemoselective Aerobic Oxidations of Sulfides and Alcohols with Potassium Nitrate and Pyridinium Tribromide or Bromine. *Synlett*. **2011**, *4*, 559-564.

[6] Casado-Sánchez, A.; Gómez-Ballesteros, R.; Tato, F.; Soriano, F.J.; Pascual-Coca, G.; Cabrera, S.; Alemán, J. Pt(II) Coordination Complexes as Visible Light Photocatalysts for the Oxidation of Sulfides Using Batch and Flow Processes. *Chem. Commun.* **2016**, *58*, 9137-9140.

[7] Renard, P.-Y., Vayron, P., Leclerc, E., Valleix, A. and Mioskowski, C. Lewis Acid Catalyzed Room-Temperature Michaelis–Arbuzov Rearrangement. *Angew. Chem. Int. Ed.* **2003**, *42*, 2389-2392.

[8] Qvortrup, K.; Rankic, D.A. MacMillan, D.W.C. A General Strategy for Organocatalytic Activation of C-H Bonds via Photoredox Catalysis: Direct Arylation of Benzylic Ethers. *J. Am. Chem. Soc.* **2014**. *136*, 626-629.

[9] Han, C.; Zhang, Z.; Xu, S.; Wang, K.; Chen, K.; Zhao, J. Palladium-Catalyzed Hiyama Coupling of Benzylic Ammonium Salts via C-N Bond Cleavage. *J. Org. Chem.* **2019**, *84*, 16308-16313.

[10] Kalola, A.G.; Prasad, P.; Mokariya, J.A.; Patel, M.P. A mild and selective Cu(II) salts-catalyzed reduction of nitro, azo, azoxy, N-aryl hydroxylamine, nitroso, acid halide, ester, and azide compounds using hydrogen surrogacy of sodium borohydride. *Synthetic Communications.* **2021**, *51*, 3565-3589.

[11] Han, C.; Buchwald, S.L. Negihi Coupling of Secondary Alkylzinc Halides with Aryl Bromides and Chlorides. *J. Am. Chem. Soc.* **2009**, *131*, 7532-7533.

[12] Amatore, M.; Gosmini, C. Synthesis of Functionalised Diarylmethanes via a Cobalt-Catalysed Cross-Coupling of Arylzinc Species with Benzyl Chlorides. *Chem. Commun.* **2008**. 5019-5021.

[13] Zhao, C.; Zha, G.-F.; Fang, W.-Y.; Rakesh, K.P.; Qin, H.-L. Construction of Di(hetero)arylmethanes Through Pd-Catalyzed Direct Dehydroxylative Cross-Coupling of Benzylic Alcohols and Aryl Boronic Acids Mediated by Sulfuryl Fluoride (SO_2F_2). *Eur. J. Org. Chem.* **2019.** *2019*, 1801-1807.

[14] Schwaller, P.; Hoover, B.; Reymond, J.-L.; Strobelt, H.; Laino, T. *Scientific Advances.* **2021**, *7*, eabe4166.

[15] Gaussian 16, Revision C.01, Frisch, M. J.; Trucks, G. W.; Schlegel, H. B.; Scuseria, G. E.; Robb, M. A.; Cheeseman, J. R.; Scalmani, G.; Barone, V.; Petersson, G. A.; Nakatsuji, H.; Li,

X.; Caricato, M.; Marenich, A. V.; Bloino, J.; Janesko, B. G.; Gomperts, R.; Mennucci, B.; Hratchian, H. P.; Ortiz, J. V.; Izmaylov, A. F.; Sonnenberg, J. L.; Williams-Young, D.; Ding, F.; Lipparini, F.; Egidi, F.; Goings, J.; Peng, B.; Petrone, A.; Henderson, T.; Ranasinghe, D.; Zakrzewski, V. G.; Gao, J.; Rega, N.; Zheng, G.; Liang, W.; Hada, M.; Ehara, M.; Toyota, K.; Fukuda, R.; Hasegawa, J.; Ishida, M.; Nakajima, T.; Honda, Y.; Kitao, O.; Nakai, H.; Vreven, T.; Throssell, K.; Montgomery, J. A., Jr.; Peralta, J. E.; Ogliaro, F.; Bearpark, M. J.; Heyd, J. J.; Brothers, E. N.; Kudin, K. N.; Staroverov, V. N.; Keith, T. A.; Kobayashi, R.; Normand, J.; Raghavachari, K.; Rendell, A. P.; Burant, J. C.; Iyengar, S. S.; Tomasi, J.; Cossi, M.; Millam, J. M.; Klene, M.; Adamo, C.; Cammi, R.; Ochterski, J. W.; Martin, R. L.; Morokuma, K.; Farkas, O.; Foresman, J. B.; Fox, D. J. Gaussian, Inc., Wallingford CT, 2016.

[16] Pedregosa, F. *et. al.* Scikit-learn: Machine Learning in Python. *JMLR*. **2011**, *12*, 2825-2830.

[17] Zahrt, A.F.; Henle, J.J.; Denmark, S.E. Cautionary Guidelines for Machine Learning Studies with Combinatorial Datasets. *ACS Comb. Sci.* **2020**, *22*, 586–591.

[18] Ruddigkeit, L.; van Deursen, R.; Blum, L.C.; Reymond, J.-L. Enumeration of 166 Billion Organic Small Molecules in the Chemical Universe Database GDB-17*. J. Chem. Inf. Model*. **2012**, *52*, 2864–2875.

[19] Blum, L.C.; Reymond, J.-L. 970 Million Druglike Small Molecules for Virtual Screening in the Chemical Universe Database GDB-13. *J. Am. Chem. Soc.* **2009**, *131*, 8732-8733.

[20] Ramakrishnan, R.; Hartmann, M.; Tapavicza, E. von Lilienfeld, O.A. Electronic Spectra from TDDFT and Machine Learning in Chemical Space. *J. Chem. Phys.* **2015**, *143*, 084111.

[21] Zahrt, A. F.; Henle, J. J.; Rose, B. T.; Wang, Y.; Darrow, W. T.; Denmark, S. E. Prediction of higher-selectivity catalysts by computer-driven workflow and machine learning. *Science.* **2019**, *363*, eaau5631.

[22] Schrödinger Release 2019-4**:** MacroModel, Schrödinger, LLC, New York, NY, 2019.
